# MicroRNA-485-5p targets keratin 17 to regulate oral cancer stemness and chemoresistance via the integrin/FAK/Src/ERK/β-catenin pathway

**DOI:** 10.1186/s12929-022-00824-z

**Published:** 2022-06-15

**Authors:** Te-Hsuan Jang, Wei-Chieh Huang, Shiao-Lin Tung, Sheng-Chieh Lin, Po-Ming Chen, Chun-Yu Cho, Ya-Yu Yang, Tzu-Chen Yen, Guo-Hsuen Lo, Shuang-En Chuang, Lu-Hai Wang

**Affiliations:** 1 Institute of Molecular Medicine, National Tsing Hua University, Hsinchu, Taiwan; 2 National Institute of Cancer Research, National Health Research Institutes, Miaoli, Taiwan; 3 Graduate Institute of Integrated Medicine, China Medical University, Taichung, Taiwan; 4 Chinese Medicine Research Center, China Medical University, Taichung, Taiwan; 5 Department of Hematology and Oncology, Ton-Yen General Hospital, Zhubei City, Hsinchu County Taiwan; 6 Department of Nursing, Hsin Sheng Junior College of Medical Care and Management, Taoyuan City, Taiwan; 7 Department of Nuclear Medicine and Molecular Imaging Center, Chang Gung Memorial Hospital and Chang Gung University, Taoyuan, Taiwan; 8 Department of Chemistry, University of Illinois at Urbana-Champaign, Urbana, IL USA

**Keywords:** Keratin 17, miR-485-5p, Integrin β4, β-catenin, Dasatinib, Cancer stemness, Chemoresistance, Oral squamous cell carcinoma

## Abstract

**Background:**

The development of drug resistance in oral squamous cell carcinoma (OSCC) that frequently leads to recurrence and metastasis after initial treatment remains an unresolved challenge. Presence of cancer stem cells (CSCs) has been increasingly reported to be a critical contributing factor in drug resistance, tumor recurrence and metastasis. Thus, unveiling of mechanisms regulating CSCs and potential targets for developing their inhibitors will be instrumental for improving OSCC therapy.

**Methods:**

siRNA, shRNA and miRNA that specifically target keratin 17 (KRT17) were used for modulation of gene expression and functional analyses. Sphere-formation and invasion/migration assays were utilized to assess cancer cell stemness and epithelial mesenchymal transition (EMT) properties, respectively. Duolink proximity ligation assay (PLA) was used to examine molecular proximity between KRT17 and plectin, which is a large protein that binds cytoskeleton components. Cell proliferation assay was employed to evaluate growth rates and viability of oral cancer cells treated with cisplatin, carboplatin or dasatinib. Xenograft mouse tumor model was used to evaluate the effect of KRT17- knockdown in OSCC cells on tumor growth and drug sensitization.

**Results:**

Significantly elevated expression of KRT17 in highly invasive OSCC cell lines and advanced tumor specimens were observed and high KRT17 expression was correlated with poor overall survival. KRT17 gene silencing in OSCC cells attenuated their stemness properties including markedly reduced sphere forming ability and expression of stemness and EMT markers. We identified a novel signaling cascade orchestrated by KRT17 where its association with plectin resulted in activation of integrin β4/α6, increased phosphorylation of FAK, Src and ERK, as well as stabilization and nuclear translocation of β-catenin. The activation of this signaling cascade was correlated with enhanced OSCC cancer stemness and elevated expression of CD44 and epidermal growth factor receptor (EGFR). We identified and demonstrated KRT17 to be a direct target of miRNA-485-5p. Ectopic expression of miRNA-485-5p inhibited OSCC sphere formation and caused sensitization of cancer cells towards cisplatin and carboplatin, which could be significantly rescued by KRT17 overexpression. Dasatinib treatment that inhibited KRT17-mediated Src activation also resulted in OSCC drug sensitization. In OSCC xenograft mouse model, KRT17 knockdown significantly inhibited tumor growth, and combinatorial treatment with cisplatin elicited a greater tumor inhibitory effect. Consistently, markedly reduced levels of integrin β4, active β-catenin, CD44 and EGFR were observed in the tumors induced by KRT17 knockdown OSCC cells.

**Conclusions:**

A novel miRNA-485-5p/KRT17/integrin/FAK/Src/ERK/β-catenin signaling pathway is unveiled to modulate OSCC cancer stemness and drug resistance to the common first-line chemotherapeutics. This provides a potential new therapeutic strategy to inhibit OSCC stem cells and counter chemoresistance.

**Supplementary Information:**

The online version contains supplementary material available at 10.1186/s12929-022-00824-z.

## Background

Oral squamous cell carcinoma (OSCC) is a subcategory of head and neck squamous cell carcinoma (HNSCC) that accounts for 90% of oral cancers and is ranked the 11th most common cancer worldwide due to its high aggressiveness with frequent local recurrences and lymph node metastases [1–3]. Despite routine therapies for oral cancers involving multi-disciplinary approaches including surgical resection combined with chemotherapy, radiotherapy or targeted therapy such as cetuximab that targets epidermal growth factor receptor (EGFR) [1, 4, 5], clinical prognostic outcome is far from satisfactory. It has been reported that both incidence and death rates for OSCC have continued to rise over the past decade [6], as 5-year survival rate for advanced OSCC patients has remained at a dismal 50% [7, 8]. To date, cisplatin or cetuximab-based chemotherapy or concurrent chemo-radiotherapy is still the standard treatment for advanced OSCC [4, 9], the development of drug resistance has become the major obstacle that frequently leads to tumor recurrence and distant metastasis [9, 10]. Immunotherapy is only beneficial to a small portion of OSCC patients with specific immunological contexture [5]. It is, therefore, important to unveil new therapeutic targets and unravel the mechanisms that underlie drug resistance and recurrence in OSCC in order to improve therapeutic regimens.

Cancer stem cells (CSCs) are a small subpopulation within a variety of tumors and have the abilities of self-renewal and differentiation, and are considered one of the most important reasons that drives drug resistance, tumor recurrence and metastasis [11, 12]. In our previous studies, we successfully enriched cancer spheroids from ovarian, triple negative breast cancer and OSCC that were shown to display characteristic hallmarks of CSCs such as retaining self-renewal ability, elevated CSC surface markers, chemoresistance and tumorigenicity [13, 14]. Multiple studies have also suggested that CSCs play a pivotal role in tumorigenesis and cancer progression in OSCC [15, 16]. Therefore, targeting CSCs may provide an effective strategy in the treatment of OSCC [17].

Keratins are epithelial-specific intermediate filament proteins with 54 functional coding genes identified that associate with integrins through plectin to stabilize hemidesmosomes for stable cell adhesion and migration [18, 19]. Recently, altered expression of several keratins including keratin 6 (KRT6), KRT14, KRT15, KRT16, KRT17 and KRT19 were reported to coordinate tumorigenesis in different types of cancers [14, 20–22]. KRT17 is upregulated in various types of tumors, including oral cancer, breast cancer, non-small cell lung cancer and cervical cancer [23–26]. It has been reported that KRT17 promotes cell proliferation and migration in OSCC and lung cancer [21, 23]. In addition, it has been shown that high levels of KRT17 expression in cervical cancer are associated with poor prognosis and cancer cell differentiation, and that KRT17 could be used as a prognostic biomarker [26–28]. Moreover, upregulation of KRT17 in esophageal cancer not only promotes cell proliferation but also increases invasion and metastasis via activation of the AKT pathway. KRT17 is significantly associated with clinical outcome of metastasis, tumor stage and 5-year survival in esophageal squamous cell carcinoma [29].

Nevertheless, the roles of keratins in mediating cancer stemness and the underlying mechanisms remain largely understudied. There is currently only one report that suggests KRT19 as a CSC marker in hepatocellular carcinoma (HCC) [22], while another recent study demonstrated that inter-keratin fusions between KRT6 and KRT14 could promote cancer stemness in OSCC [20]. Previously, we had established highly invasive oral cancer cell lines from lung metastatic tumor nodules and identified miR-365-3p/KRT16 and miRNA-491-5p/G-protein-coupled receptor kinase-interacting protein 1 as crucial modulators for regulating cellular invasion and metastasis in OSCC [14, 30]. In fact, the miR-365-3p/KRT16 signaling also pertains the ability to regulate cancer stemness and drug resistance via interaction with the β5-integrin/c-Met pathway in promoting tumorigenesis of OSCC. In the present study, we report another keratin member, KRT17, and unravel the role of KRT17 in modulating cancer stemness and chemoresistance of OSCC through association with integrin β4 and several downstream signaling molecules including FAK, Src and β-catenin in OSCC.

## Methods

### Clinical specimens

A cohort of 45 OSCC specimens obtained from Chang Gung Memorial Hospital (CGMH) at Linkou, Taiwan, was used for cDNA microarray and data analyses in accordance of Institutional Review Board (IRB) protocol from CGMH. The study protocol (100-4440A3) was approved by the Research Ethics Committee of CGMH. Commercial tissue array slides containing 145 OSCC tissues were purchased from US Biomax, Inc. (Rockville, MD, USA). In addition, 24 oral tumor tissue samples were obtained from the Oral and Maxillofacial Surgery Unit, Chi Mei Medical Center (CMMC), Tainan, Taiwan, and Department of Public Health and Environmental Medicine, School of Medicine, College of Medicine, Kaohsiung Medical University (KMU), Taiwan. These 24 specimens were acquired and analyzed in accordance with protocols approved by the Institutional Review Board in our previous study [30].

### Cell lines and transfections

Oral squamous cancer cell lines and derivative cell lines utilized in the current study including OC3, OC3IV, CGHNC9 (C9) and C9IV3 were described in our previous study [14]. The human tongue and oral squamous carcinoma cell lines HSC3 and OECM1 were commercially acquired from ATCC. The cells were cultured in DMEM with 10% fetal bovine serum (Invitrogen, Carlsbad, CA, USA) in a humidified atmosphere at 37 °C and 5% CO_2_. siRNAs targeting KRT17, plectin, integrin β4, β-catenin and KRT6 were purchased from MDBio, Inc. (Taipei, Taiwan). The siRNAs sequences are listed in Table S1. C9IV3 and HSC3 cells were transfected with specific miRNAs individually using the Lipofectamine 2000 (Invitrogen) for 48 h before harvest for experimental assays.

### Microarray analysis

Total RNAs were extracted from the parental OC3 and highly invasive OC3IV cells for gene expression analysis using Affymetrix GeneChip system (Affymetrix, Santa Clara, CA, USA) and the microarray statistical analysis was performed by the Microarray Core Laboratory at the National Health and Research Institute (NHRI, Taiwan).

### Plasmid construction

KRT17 and β-catenin coding sequences were individually cloned into the pcDNA3.1 plasmid. Wild-type KRT17-3′ untranslated region (UTR) was cloned into the pGL3-control plasmid, while mutant KRT17-3′UTR was subsequently generated by site-directed mutagenesis. Stable transfection of C9IV3 cells with scrambled or KRT17 shRNA plasmid was conducted using a lentivirus-mediated RNA interference system (pLKO.1, pCMV-△R8.91, pMD.G) according to the manufacturer's instructions from the National RNAi Core Facility of Academia Sinica (Taipei, Taiwan). The KRT17 shRNA plasmid was also purchased from the National RNAi Core Facility of Academia Sinica (Taipei, Taiwan): (TRCN0000318921). Puromycin at 1 µg/mL was used to treat the lentivirus infected cell population for selection of drug resistant transfectants. The transfection efficacy was confirmed by Western blotting. Primer sequences are described in the Additional file 1: Table S1.

### Sphere-forming assay

Sphere-forming assay was performed as previously described [13, 14]. Briefly, C9IV3 and HSC3 cells were transfected with plasmids containing siCon or siKRT17 for 24 h, or miR-Con, miR-485-5p, miR-485-5p + KRT17 for 48 h, or in combination with 1 μM dasatinib (Selleckchem, Houston, Texas, USA) treatment for 48 h prior to sphere forming assay. Transfected and/or treated cells were cultured in stem cell selective medium at cell density of 1 × 10^5^ cells per well with 3 ml of PSGro hESC/iPSC growth medium (System Biosciences, Palo Alto, CA, USA) in Costar ultra-low attachment 6-well plates (Corning, Oneonta, NY, USA). The total numbers of spheres formed were counted under microscope at day 10.

### Cell proliferation assay

MTS assay (Promega, Madison, WI, USA) was performed according to the manufacturer’s protocol. For cellular drug sensitivity assays, C9IV3 and HSC3 cells were transfected with siRNA (siCon or siKRT17), or miRNA (miR-Con, miR-485-5p, or miR-485-5p + KRT17) plasmids for 24 h. In drug treatment experiments, C9IV3 and HSC3 cells were treated with 1 μM dasatinib for 48 h before seeding at a density of 8 × 10^3^ cells/100 μl/well in 96-well plates. Subsequently, they were incubated in media with different concentrations (ranging from 0 to 250 μM) of cisplatin or carboplatin (Selleckchem). After 48 h of treatments, MTS were added to each well and incubated for 3 h before optical density values were measured at wavelength 490 nm.

### Preparation of cytoplasmic/nuclear proteins

Nuclear and cytoplasmic proteins were prepared using a Nuclear and Cytoplasmic Protein Extraction Kit (Active Motif, Carlsbad, CA, USA) according to the manufacturer's instructions.

### Western blot assay

Proteins from transfected or treated cells were extracted and separated by SDS–PAGE before transferring to a nitrocellulose membrane, which was subsequently exposed to the appropriate primary antibody before detection using the horseradish peroxidase conjugated secondary antibody. Enhanced chemiluminescence (ECL) images were developed using ECL developer (Millipore, Darmstadt, Germany). Primary and secondary antibodies used are listed in the Additional file 1: Table S2.

### Reverse transcription quantitative polymerase chain reaction (RT-qPCR)

RT-qPCR was used to detect the mRNA and microRNA expression in total RNAs extracted from cultured cells using TRIZOL reagent (Invitrogen). Reverse transcription of the total RNAs to cDNA using reverse transcriptase from TOYOBO (Osaka, Japan). Actin was used as an internal control. For miRNA detection, a stem-loop RT primer was designed for microRNA hybridization and RNU6B was used for normalization. PCR was performed in a Bio-Rad CFX96 real-time PCR detection system using SyBr Green reagents from KAPA Biosystems (Wilmington, MA, USA). PCR primers used are described in the Additional file 1: Table S3.

### Proximity ligation assay

C9IV3 cells were fixed with 3.7% paraformaldehyde and blocked with the blocking solution supplied by the Duolink PLA kit (Olink Bioscience, Uppsala, Sweden) according to the manufacturer’s instructions. Briefly, the fixed cells were incubated with anti-KRT17 antibody and anti-Plectin antibody overnight. After multiple washings, cells were incubated successively with PLA probes, ligation solution and amplification solution at 37 °C. Cover-slips were mounted and the images were examined using Leica TCS SP5 confocal microscope (Leica Microsystems, Mannheim, Germany).

### 3′-Untranslated region (3′UTR) luciferase reporter assay

pGL3-KRT17-3′UTR-wt or pGL3-KRT17-3′UTR-mt was co-transfected with miR-485-5p into C9IV3 and HSC3 cells. Renilla vector was co-transfected as an internal control for normalization. After transfection for 48 h, dual-luciferase reporter assay system (Promega) was used for luciferase activity detection according to the manufacturer’s instructions.

### Immunohistochemistry (IHC) and immunofluorescence assays

IHC was performed using antibodies specifically against KRT17, ITGB4, Active (non-phosphorylated) β-catenin, CD44 and EGFR individually in NHRI Pathology Core Laboratory. Secondary antibodies were then applied based on manufacturer’s suggestion. Immunoreactivity was visualized by using Vectastain ABC kit (Vector Laboratories, Burlingame, CA, USA). Sections were photographed by Leica DM2500 (Leica Microsystems, Wetzlar, Germany). The KRT17 IHC scores for specimens were as follows: negative or weakly positive (1), moderately positive (2) and strongly positive (3). C9IV3 cells were seeded on glass cover-slips in 6-well plates. Cells were transfected with siCon or siKRT17 for 48 h. Cells were fixed with 3.7% paraformaldehyde and blocked with 5% BSA in PBS for 30 min and incubated with anti-KRT17 overnight. After repeated washings, cells were incubated with AlexaFluor 488-conjugated secondary antibody (Invitrogen) as listed in Additional file 1: Table S2 for fluorescent detection and analysis.

### Mouse xenograft model and tumor growth assay

1 × 10^5^ cells of C9IV3 (stable shCon- or shKRT17-transfected cells) were suspended in 100 μl PBS and injected subcutaneously into the upper right flank of CB17 SCID mice (BioLasco, Taipei, Taiwan). Tumor-bearing mice from each group (n = 10) were randomly further divided into two subgroups (n = 5 per group) and intravenously administered with PBS or cisplatin (5 mg/kg) twice a week. Tumor size was measured once a week. At the end of the treatment period, the mice were sacrificed and the tumors were excised, fixed, paraffin embedded and sectioned for IHC staining and analysis. All the mouse experimental procedures were carried out according to the protocol approved by Institutional Animal Care and Use Committee of NHRI.

### Survival rate

The Kaplan–Meier (KM) plotter survival analysis was used to assess whether KRT17 expression affected the clinical outcome of Head-neck cancer patients in terms of survival. A total of 307 male Caucasian patients were analyzed from Oncomine database (http://www.oncomine.org/) [31]. KM plotter survival analysis was also performed using the cDNA microarray data from local cohort of 45 OSCC tumor specimens from CGMH.

### Statistics

The GraphPad Prism software (San Diego, CA, USA) was used for graph plotting and statistical analyses. Student t-tests were used to determine differences between experimental groups including drug resistance in dose-dependent growth inhibition or the growth of xenograft tumors between experimental groups. The differences in the expression levels of KRT17 in the OSCC tissues with different pathologic stages were calculated using Fisher’s exact probability test. Statistical significance was accepted with P < 0.05.

## Results

### Identification of KRT17 as a prognosis biomarker for OSCC

**Fig. 1:**
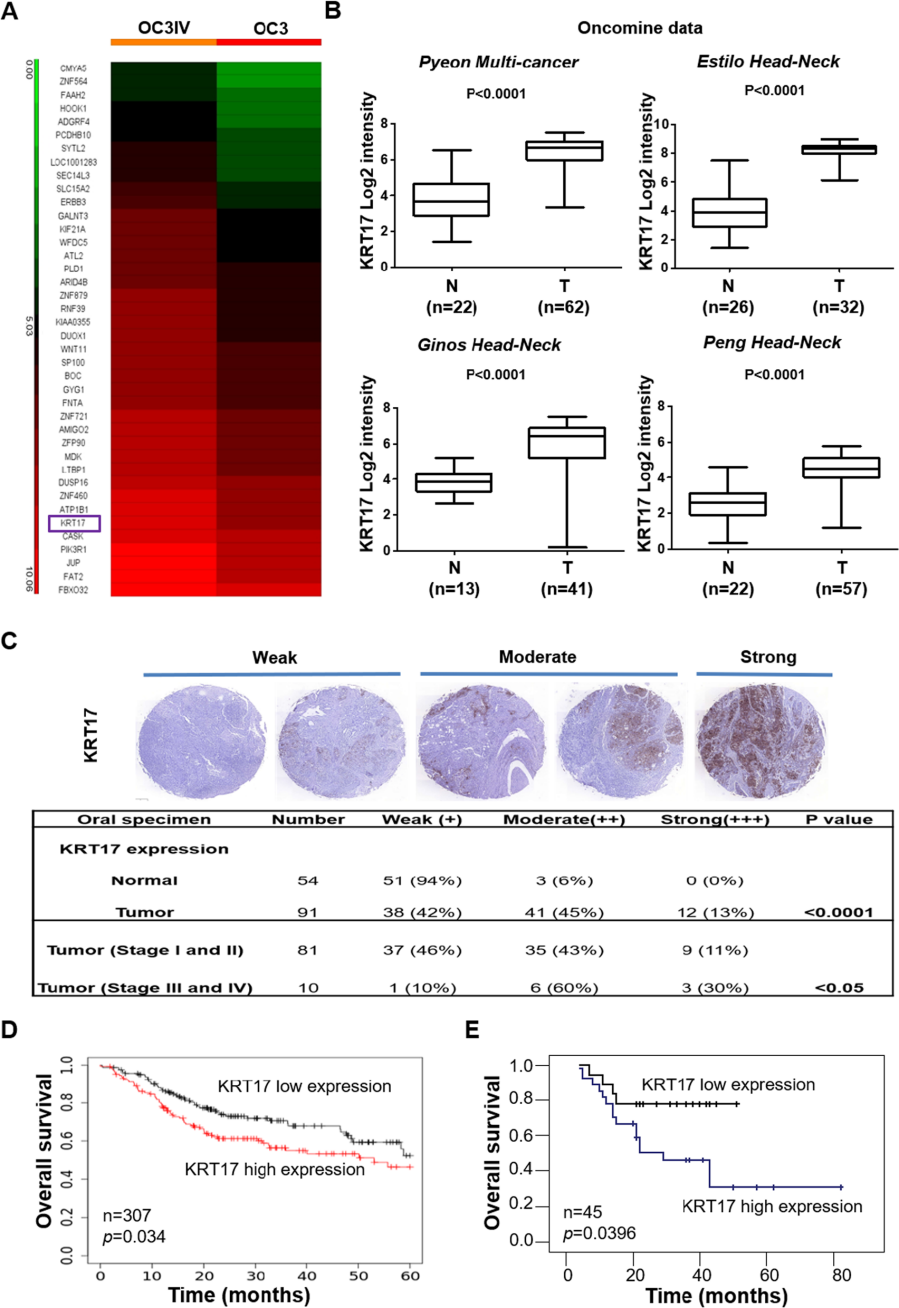
The clinical importance of KRT17 as a prognostic marker in OSCC. **A** Heatmap analysis of differentially-expressed genes from cDNA microarrays data that were derived from OC3IV (left panel) and OC3 (right panel) OSCC cells. **B** Datamining analysis of KRT17 mRNA expression in the adjacent normal tissues (N) and OSCC tumor tissues (T) from Oncomine database. **C** Representative images of IHC staining for the KRT17 protein expression were shown using 3-level pathological ranking of weak, moderate and strong from a set of commercial tissue arrays containing a total of 145 normal and OSCC tissues (top panel). The bottom panel shows a table summarizing statistically the expression differences of KRT17 between normal and OSCC tissues with different pathological stages at I/II or III/IV. **D** KM plotter survival analysis was performed to examine KRT17 gene expression in a cohort of 307 Head-neck cancer patients (Oncomine database). **E** Correlation of KRT17 expression to overall survival rates was determined by performing cDNA microarray with 45 OSCC tumor specimens as described in “Methods”

The lack of biomarkers for effective prognosis of OSCC is still an unmet need [32]. To identify novel OSCC biomarkers that implicate better treatment options and prognosis, we utilized microarray analysis to investigate differential gene expression in the highly invasive OC3IV cells. The OC3IV cell line was obtained by in vivo injection of OC3 cells into the tail vein of C.B-17 severe combined immunodeficiency (CB17-SCID) mice, followed by isolation of tumor cells grown from lung metastases as described in our previous publication [14]. By comparison with parental OC3 cells, KRT17 was identified as one of the top differentially-expressed genes (DEGs) (Fig. 1A). We assessed the clinical significance among the top DEGs using Oncomine database [31], and found that significantly increased KRT17 mRNA expression was closely associated with tumor tissues in multiple types of cancer including HNSCC when compared to their adjacent normal tissues (Fig. 1B). To access whether KRT17 is clinicopathologically correlated with OSCC, we examined protein expression of KRT17 with tissue arrays that contained 54 normal and 91 OSCC tumor tissues by IHC staining. As shown in Fig. 1C, the KRT17 protein expression was significantly increased in OSCC tumors when compared to the normal counterparts. Importantly, further analysis based on clinicopathological grade of the 91 OSCC tumor tissues revealed that the KRT17 protein expression level was significantly higher in tumors at stage III/IV than those at stage I/II (Fig. 1C). Of note, there were 90% of tumor tissues scored at moderate to strong expression of KRT17 from the stage III/IV specimens as compared to only 54% of tumor tissues were scored from the stage I/II specimens. In line with those observations, the KM plotter survival analysis of a 307 head-neck cancer cohort revealed a significant association of higher KRT17 levels with worse overall survival (Fig. 1D). Further, we analyzed a set of cDNA microarray data containing 45 OSCC specimens from a local Taiwanese cohort. The results indicated that patients with higher KRT17 expression had significantly worse survival (Fig. 1E). These findings implicated that elevated KRT17 expression was strongly associated with more advanced clinicopathological stages and worse survival in OSCC patients.

### KRT17 regulates cancer stemness and epithelial mesenchymal transition (EMT) properties in OSCC

**Fig. 2:**
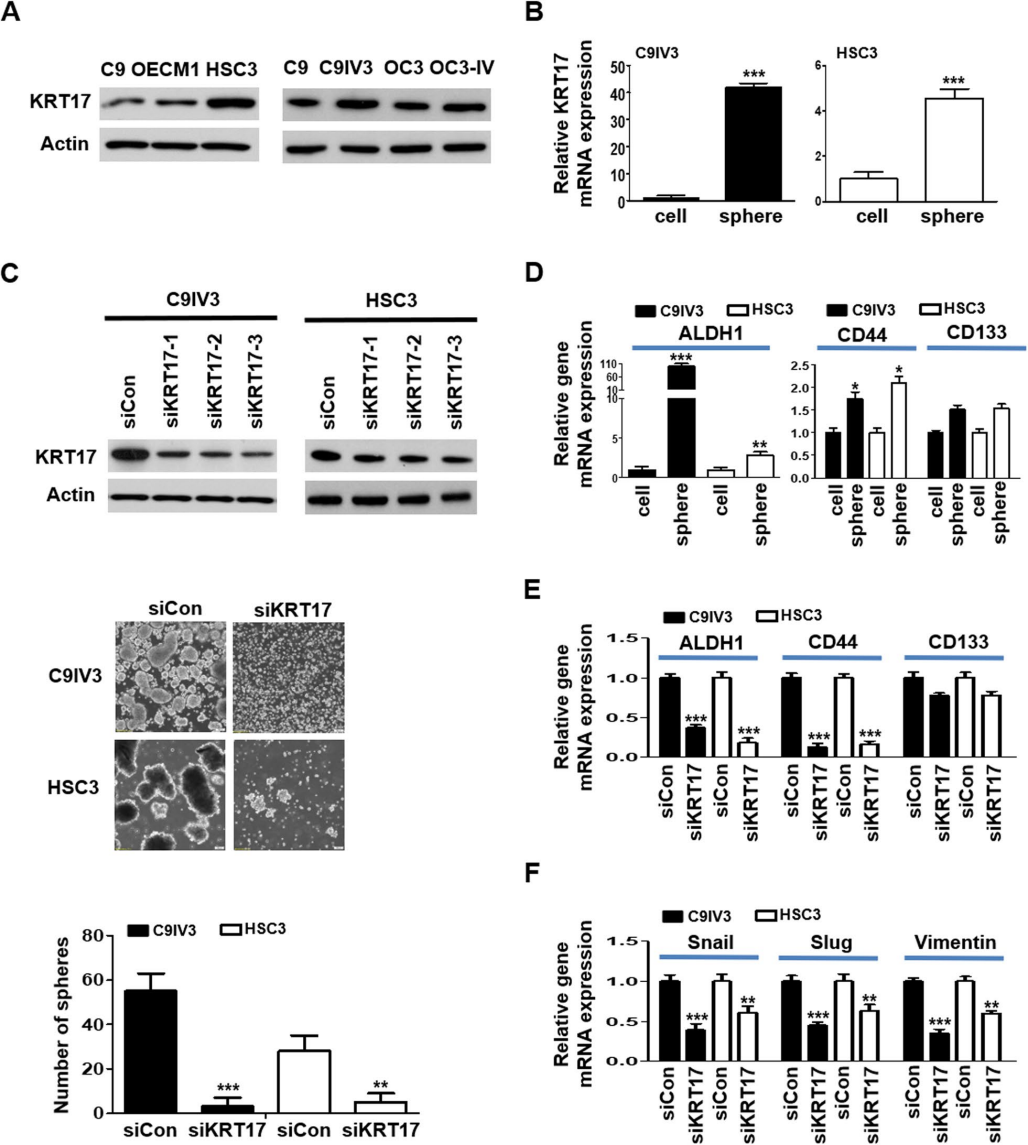
Silencing of KRT17 downregulates cancer stemness in invasive OSCC. **A** KRT17 protein expressions in different OSCC cell lines were assessed by immunoblotting. **B** KRT17 mRNA expressions in the spheres formed from C9IV3 or HSC3 cells were analyzed by comparisons to their parental non-sphere C9IV3 or HSC3 cells, respectively, using qPCR. **C** Top, Protein expressions of KRT17 in C9IV3 and HSC3 cells transfected with Control-siRNAs (siCon) or three specific KRT17-siRNA plasmids (siKRT17-1, -2, -3) were assessed by immunoblotting. Middle, sphere-forming abilities of C9IV3 and HSC3 cells transfected with siCon or siKRT17 (siKRT17-3). Bottom, quantitative analysis of the number of spheres formed. **D** ALDH1, CD44 and CD133 expressions in spheres enriched from C9IV3 or HSC3 cells versus non-sphere cells were determined by qPCR. **E** ALDH1, CD44 and CD133 expressions in the C9IV3 or HSC3 spheres and non-sphere cells that had been transfected with siCon or siKRT17 were determined by qPCR. **F** Expressions of EMT markers Snail, Slug and Vimentin in the same spheres or non-sphere cells as in **D**, **E**. were determined by qPCR. Data are presented as mean ± SD (*p < 0.05, **p < 0.01 and ***p < 0.001)

To date, KRT19 remains the only keratin that has been reported to be one of the cancer stemness markers including CD133, EpCAM and c-kit in HCC [33]. To further elucidate the functional role of KRT17 in OSCC, we examined protein expression level of KRT17 in several commonly used OSCC cell lines. The C9IV3 cell line was obtained by in vivo injection of C9 cells into the tail vein of CB17-SCID mice, followed by isolation of tumor cells grown from lung metastases as described in our previous publication [14]. Immunoblotting results showed that HSC3 expressed the highest level of KRT17 among the OSCC cell lines examined, while the more invasive C9IV3 cells expressed a higher level of KRT17 than its parental C9 cells (Fig. 2A). To investigate whether KRT17 is involved in regulating cancer stemness in OSCC, we performed sphere-forming assay and inspected for changes in KRT17 expression. As shown by RT-qPCR assay, KRT17 mRNA expression was significantly elevated in the OSCC spheres formed from invasive C9IV3 and HSC3 cells as compared to non-sphere cells (Fig. 2B). Next, three siRNA specifically targeting KRT17 were designed to reduce the expression of KRT17 in HSC3 and C9IV3 cells, and the results showed that siKRT17-3 had the highest gene silencing efficacy in both C9IV3 and HSC3 cells and thus was utilized in subsequent experiments (Fig. 2C). Firstly, we utilized siKRT17-3 to investigate if the silencing of KRT17 could impact on cancer properties such as proliferation and colony formation of OSCC cells. The results demonstrated that C9IV3 cells transfected with siKRT17-3 grew at a significantly slower rate than those transfected with the siRNA control (siCon), while colony-forming ability was also significantly reduced when KRT17 was decreased (Additional file 1: Fig. S1A, B). Supporting those observations, specific inhibition of KRT17 markedly reduced sphere-forming ability of both C9IV3 and HSC3 cells (Fig. 2C). Since ALDH1, CD44 and CD133 have been well-recognized as CSC hallmarks in OSCC [34, 35], we assessed whether targeting KRT17 could impact on the expression of these stemness markers. Our data showed that ALDH1 and CD44 were both significantly up-regulated in the spheres enriched from C9IV3 and HSC3 cells when compared to the non-sphere cells, whereas a mild increase in CD133 was observed (Fig. 2D). In contrast, the expression of ALDH1 and CD44 were both significantly down-regulated in the spheres when KRT17 expression was reduced (Fig. 2E) suggesting a potent regulatory role of KRT17 in OSCC stemness. Moreover, EMT has been considered a key process of the regulation of cancer stemness [36–38] involved in tumor progression and resistance to therapeutics. We thus examined whether regulating KRT17 expression could also affect essential EMT markers such as Snail, Slug and Vimentin. As shown in Fig. 2F, KRT17-silencing resulted in significantly reduced expression of Snail, Slug and Vimentin in both C9IV3 and HSC3 cells (Fig. 2F). Reduced expression of EMT markers mediated by KRT17-silencing was manifested in significantly decreased migratory and invasive abilities of C9IV3 cells (Additional file 1: Fig. S1C). These findings suggest a potent role of KRT17 in the regulation of OSCC EMT and cancer stemness.

### KRT17 associates with plectin to regulate integrin/FAK/Src/ERK/β-catenin signaling in OSCC

**Fig. 3:**
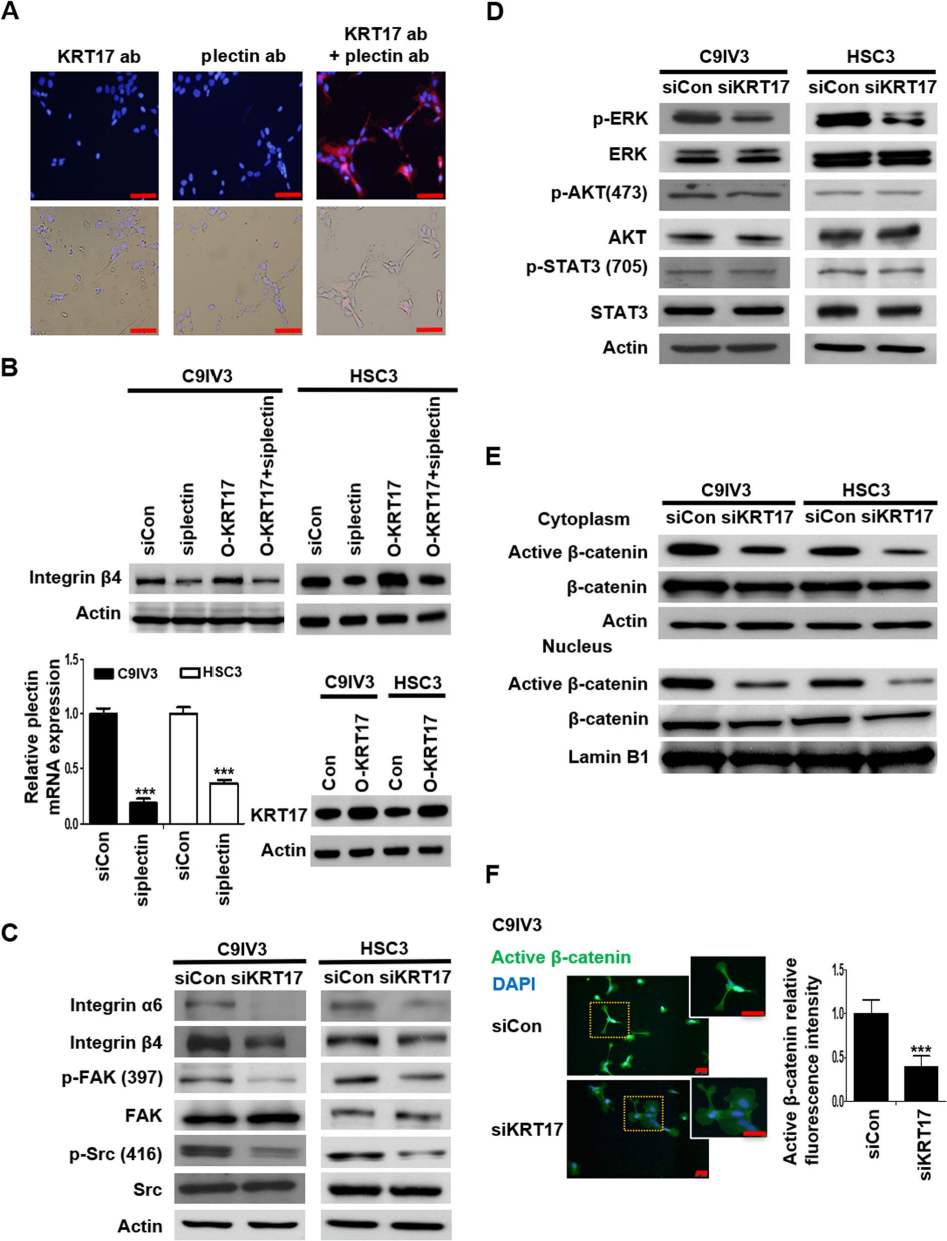
KRT17 associates with the plectin-integrin β4 complex to activate downstream FAK/Src/ERK/β-catenin signaling pathway. **A** Molecular proximity between KRT17 and plectin in C9IV3 cells was analyzed by PLA as described in the Methods. Protein complexes under examination were visualized as red dots in fluorescence and bright field images; DAPI-stained cell nuclei are shown in blue. Scale bar shown is 100 μm. **B** ITGB4 protein expressions in C9IV3 and HSC3 cells that had been transfected with siCon, si-plectin, O-KRT17 (KRT17 overexpressing plasmid) or si-plectin + O-KRT17 were determined by immunoblotting (top). qPCR analysis was used to assess mRNA expression of plectin in C9IV3 and HSC3 cells transfected with siCon or si-plectin (bottom left); while immunoblotting was used to assess KRT17 protein expression in C9IV3 and HSC3 cells that had been transfected with empty control plasmid (Con) or O-KRT17 (bottom right). Data are presented as the mean ± SD (***p < 0.001). **C–E** Integrin α6, integrin β4, p-FAK, FAK, p-Src, Src, p-ERK, ERK, p-AKT, AKT, p-STAT3, STAT3, active β-catenin (unphosphorylated) and β-catenin protein expressions in C9IV3 and HSC3 cells transfected with siCon or siKRT17 were determined by immunoblotting with antibodies specifically recognizing these proteins. **F.** Immunofluorescent staining for active β-catenin (green) was conducted to examine the influence of KRT17-silencing on β-catenin in C9IV3 cells. Scale bar shown is 20 μm. Histograms show quantification of the fluorescence intensity for the expression of Active β-catenin. Data are presented as the mean ± SD (***p < 0.001)

The implication of KRT17 in stemness regulation of OSCC prompted us to elucidate the underlying mechanisms. Keratins are well-known interacting partners with plectin, which has been reported to bind with integrin α6β4 and act as a dominant driver of malignant tumorigenesis via diverse cellular activities including cancer cell proliferation, migration and invasion [39]. We utilized Duolink PLA to determine the protein–protein interaction between KRT17 and plectin. The result displayed prominent red fluorescent signals constituted by KRT17 and plectin antibodies, reflecting the two proteins were in close proximity of less than 40 nm (Fig. 3A). To explore potential functional consequences that may be elicited by KRT17/plectin interaction, expression of integrin β4 and α6 was examined by siRNA-mediated depletion of plectin (si-plectin) and/or overexpression of KRT17 (O-KRT17) in C9IV3 and HSC3 cells. The results demonstrated that plectin-silencing resulted in significantly downregulated protein expression of integrin β4 level, whereas KRT17 overexpression led to upregulation of integrin β4, which was reversed when plectin was silenced in the KRT17-overexpressing cells (Fig. 3B). Efficacies of si-plectin knockdown and KRT17 overexpression in C9IV3 and HSC3 cells were verified by RT-qPCR analysis and immunoblotting (Fig. 3B, bottom left and bottom right, respectively). Of note, the reduction of integrin β4 in plectin-depleted cells was found to be lysosome-dependent instead of proteasome-dependent as Baflomycin-A1 (B.A.), but not MG132, treatment in C9IV3 or HSC3 cells rescued the reduced integrin β4 protein expression (Additional file 1: Fig. S2), Since the depletion of plectin and overexpression of KRT17 could differentially regulate the expression of integrin β4, we proceeded to investigate if KRT17 could impact on integrin β4-mediated signaling events. As shown in Fig. 3C, silencing of KRT17 led to significant reduction in not only integrin β4 but also integrin α6 in both C9IV3 and HSC3 cells (Fig. 3C). Integrin α6β4, a receptor for laminin 332, is associated with malignant progression and survival in various cancers due to its reported ability to activate multiple signaling cascades that contribute to cell growth, survival, invasion and metastasis [40]. In line with a previous report indicating that integrin α6β4 was able to activate FAK/Src signaling cascade [41, 42], our result showed depletion of KRT17 in these invasive OSCC cells resulted in significantly reduced phosphorylation levels of FAK(Tyr397) and Src(Tyr416) (Fig. 3C). Since Src is known to mediate signaling crosstalk between receptor tyrosine kinases and related downstream signaling effectors such as extracellular signal-regulated kinase (ERK), AKT and STAT3 via integrins [43, 44], we next assessed effect of KRT17-silencing on these signaling molecules. Our data demonstrated that depletion of KRT17 greatly suppressed the phosphorylation of ERK but not AKT or STAT3 (Fig. 3D). As Wnt/GSK3β/β-catenin axis is often reported to be activated by Src and ERK and results in nuclear translocation of β-catenin and tumor progression [42, 44, 45], we then examined subcellular localization of β-catenin and found that both cytoplasmic and nuclear active (unphosphorylated) β-catenin was markedly reduced upon KRT17 depletion (Fig. 3E). Similar observation was obtained by immunofluorescence staining, in which expression of active β-catenin was markedly reduced in both cytoplasm and nucleus of KRT17-depleted cells (Fig. 3F). We then conducted further experiments to confirm the interplay between KRT17 and the integrin β4/Src/ERK/β-catenin signaling cascade. Depletion of integrin β4 caused suppression of p-FAK and p-Src whereas overexpression of KRT17 increased both p-FAK and p-Src (Additional file 1: Fig. S3B). KRT17 overexpression-mediated increase of p-FAK and p-Src was reversed by knockdown of integrin β4 (Additional file 1: Fig. S3B). The efficacy of specific integrin β4 siRNA (siITGB4) was examined by immunoblotting analysis (Additional file 1: Fig. S3A). Moreover, siKRT17 resulted in significant reduction in the phosphorylation level of ERK (Fig. 3D). We then checked if ERK affected the expression of β-catenin. ERK inhibitor (U0126) significantly reduced the active β-catenin expression, while KRT17 overexpression increased the active β-catenin, which could be specifically reduced by ERK inhibitor. The efficacy of the specific ERK inhibitor was verified by immunoblotting analysis (Additional file 1: Fig. S4A). These data support that KRT17 elicited the ERK-β-catenin signaling axis (Additional file 1: Fig. S4B). Taken together, the above findings demonstrated that KRT17 associated with plectin to regulate integrin/FAK/Src/ERK/β-catenin signaling in OSCC.

### KRT17 regulates stemness markers CD44 and EGFR via activation of integrin/Src/β-catenin signaling cascade

**Fig. 4:**
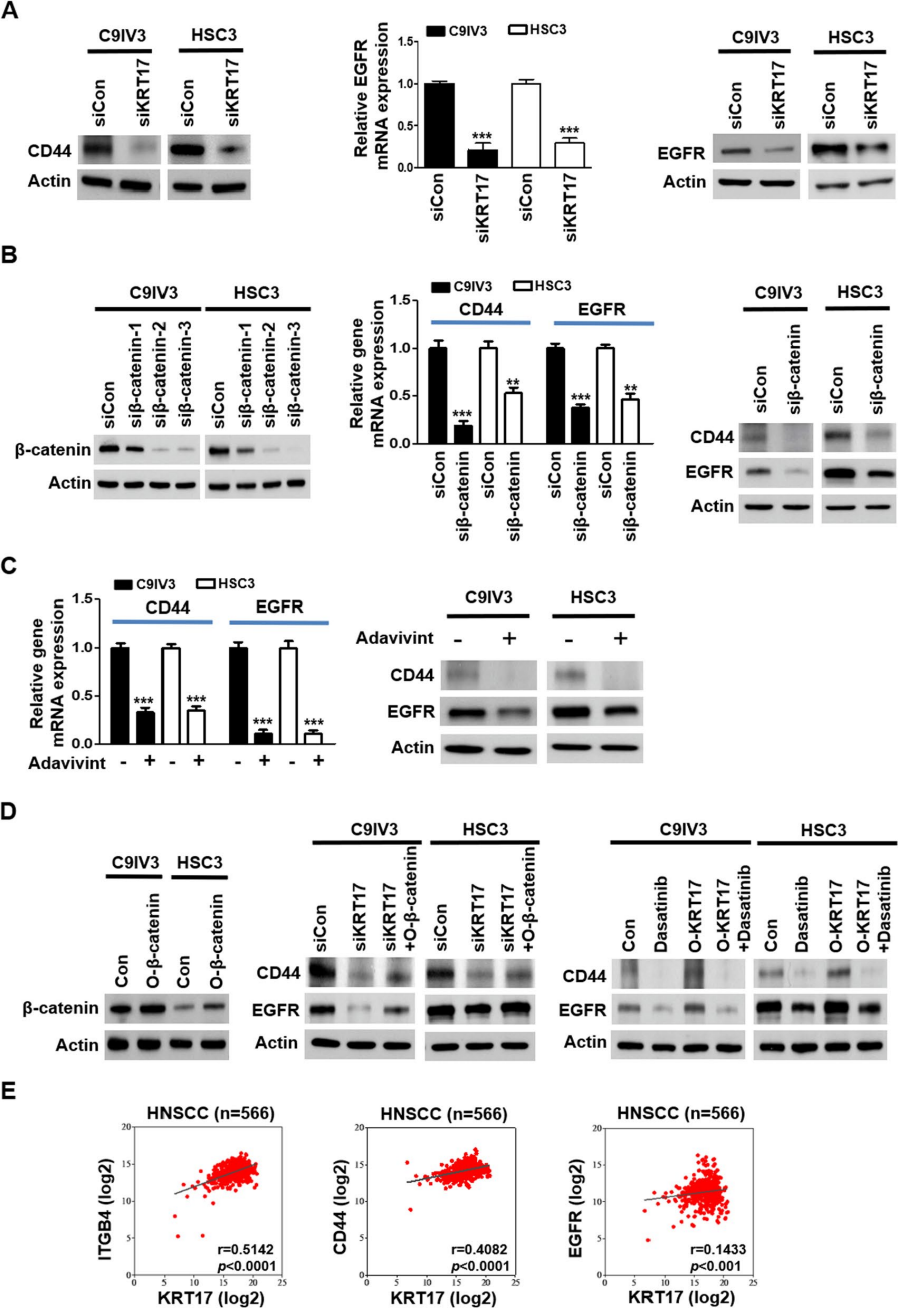
KRT17 regulates CD44 and EGFR expression via activations of integrin/Src/β-catenin signaling cascade. **A** CD44 protein expression in C9IV3 and HSC3 cells transfected with siCon or siKRT17 was determined by western blotting (left panel), and EGFR expression was assessed by qPCR and western blotting (middle and right panels). **B** Left, Western blotting was used to assess the efficacy of three siRNAs (siβ-catenin-1, -2, -3) designed to specifically silence β-catenin in C9IV3 and HSC3 cells (left panel). Siβ-catenin-2 (siβ-catenin) that caused most effective β-catenin suppression was utilized to examine mRNA and protein expression of CD44 and EGFR in C9IV3 and HSC3 cells (middle and right). **C** The canonical Wnt/β-catenin inhibitor, adavivint (1 μM), was used to treat C9IV3 and HSC3 cells for 48 h before analyzing expressions of CD44 and EGFR at both mRNA and protein levels. **D** Expression efficiency of the β-catenin cDNA plasmid (*O*-β-catenin) was determined at protein level (left panel) prior to combinatorial treatment with either siKRT17 (middle panel) or dasatinib (right panel) for assessing their impacts on CD44 and EGFR expression in C9IV3 and HSC3 cells. Cells were transfected with the indicated siRNA or plasmids for 24 h, and then treated with 1 μM dasatinib for 48 h. **E** Correlation between the gene expression levels of KRT17 and ITGB4, CD44 or EGFR in clinical HNSCC specimens using TCGA database (n = 566)

Higher levels of cytoplasmic/nuclear translocation of β-catenin has been reported to associate with oral dysplasia, higher invasive growth, risk of metastasis, recurrence rate and shorter survival [46, 47]. The ability of β-catenin in upregulating stemness markers including CD44 and epidermal growth factor receptor (EGFR) in OSCC and HNSCC have also recently been documented [48–50], prompting us to investigate whether EGFR and CD44 were also regulated by KRT17-mediated signaling cascade of integrin/Src/ERK/β-catenin. In line with reduced mRNA expression of CD44 and ALDH1, protein expression of CD44 and ALDH1 were clearly suppressed when KRT17 was depleted (Fig. 4A; Additional file 1: Fig. S5A, respectively) in both C9IV3 and HSC3 cells, while KRT17 suppression also downregulated EGFR at both transcriptional and translational levels (Fig. 4A). Next, siRNA that effectively abolished β-catenin protein levels were utilized to examine direct impact of β-catenin on CD44 and EGFR. Our results demonstrated that β-catenin suppression resulted in markedly reduced CD44 and EGFR at both mRNA and protein levels (Fig. 4B) but not ALDH1 (Additional file 1: Fig. S5B) in C9IV3 and HSC3 cells. To validate the correlation between β-catenin and CD44/EGFR, we employed a canonical Wnt/β-catenin inhibitor, adavivint, which consistently resulted in significant repressions of CD44 and EGFR at both mRNA and protein expression levels (Fig. 4C). Further, these findings were consolidated by ectopic expression of β-catenin in KRT17-silenced C9IV3 and HSC3 cells (Fig. 4D, left), as overexpression of β-catenin (*O*-β-catenin) was able to rescue the reduced protein expressions of CD44 and EGFR caused by KRT17-silencing (Fig. 4D, middle). Furthermore, the role of Src in activating β-catenin thereby enhancing CD44 and EGFR levels was evaluated by treating KRT17-overexpressed C9IV3 and HSC3 cells with clinical Src inhibitor, dasatinib. Our results demonstrated that O-KRT17-induced CD44 and EGFR expressions were markedly reduced by dasatinib (Fig. 4D, right), suggesting KRT17-mediated OSCC stemness regulation via integrin/FAK/Src/ERK/β-catenin signaling cascade, which could be of clinical significance. In fact, results from the TCGA HNSCC database (generated by the TCGA Research Network: https://www.cancer.gov/tcga) revealed significant positive correlation between KRT17 expression with those of ITGB4, CD44 and EGFR (Fig. 4E).

### miR-485-5p targets KRT17 and regulates OSCC sphere formation and drug resistance

**Fig. 5:**
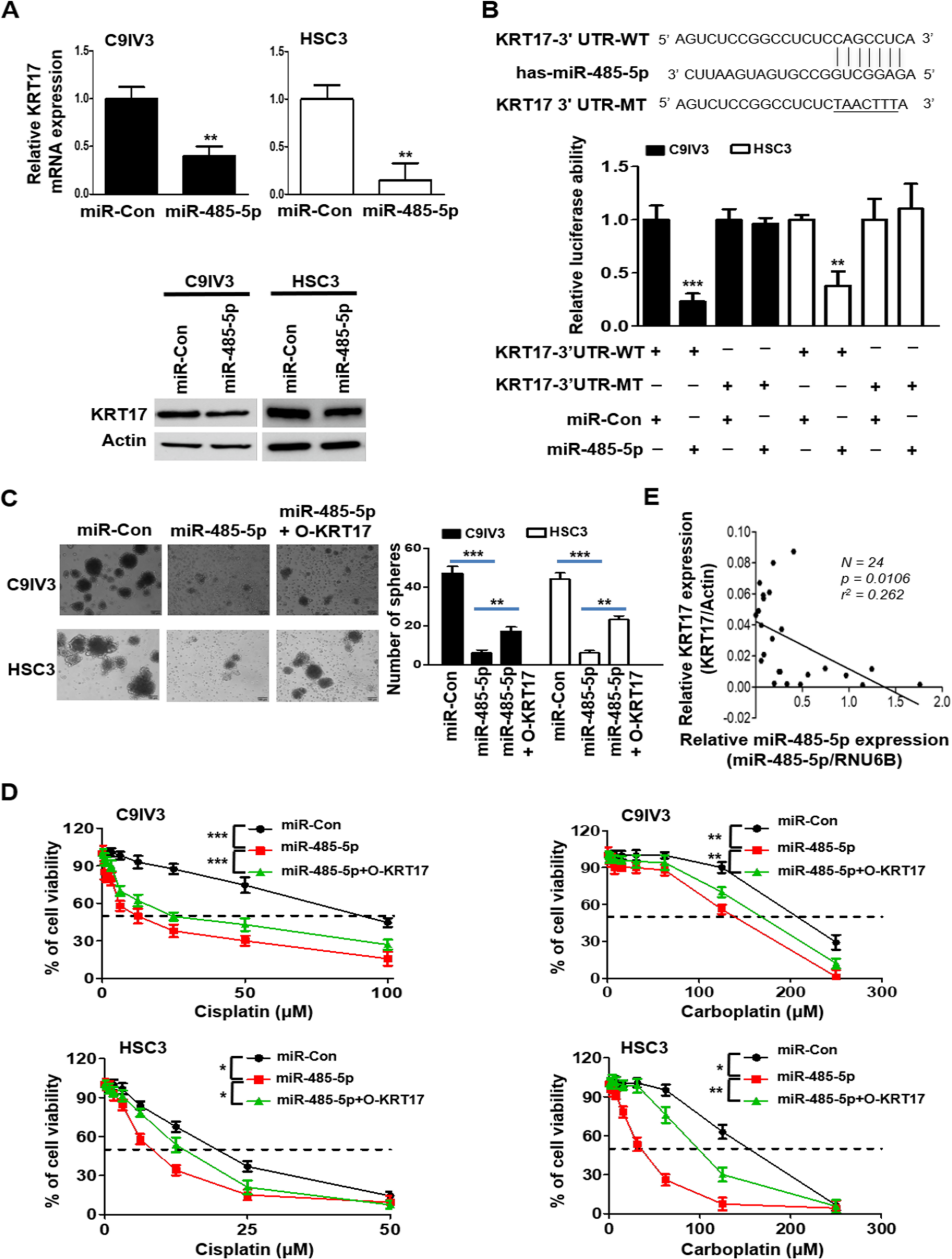
miR-485-5p suppresses KRT17 expression and sphere formation and its effect on drug sensitivity of OSCC. **A** qPCR and immunoblotting were used to assess targeting effects of miR-485-5p on KRT17 mRNA (top panel) and protein (bottom panel) expression in C9IV3 and HSC3 cells in comparison with the miR-Con. **B** Sequence alignment of miR-485-5p binding site within the KRT17 3′-UTR (top panel). Luciferase activity assays from C9IV3 and HSC3 cells transfected with wild-type (WT) or mutant (MT) KRT17 3′-UTR reporter constructs in the presence of miRNA control (miR-Con) or miR-485-5p expression plasmids (bottom panel). **C** Sphere-formation assays using C9IV3 and HSC3 cells transfected with miR-Con, miR-485-5p or miR-485-5p + O-KRT17 (left panel). Quantitative analysis on the number of spheres formed is shown in the right panel. **D** Dose-dependent cellular growth assays using C9IV3 or HSC3 cells transfected with miR-Con, miR-485-5p or miR-485-5p + O-KRT17 plasmid in combination of treatment with cisplatin (0–100 μM) or carboplatin (0–250 μM). Data are presented as the mean ± SD (*p < 0.05, **p < 0.01 and ***p < 0.001). **E** Correlation analysis between miR-485-5p and KRT17 RNA levels in 24 OSCC tumor specimens from Kaohsiung Medical University, Taiwan

Oncogenes and stemness regulatory genes are increasingly reported to be modulated by binding of microRNAs (miRNAs) to the 3′UTR sequences that leads to mRNA degradation or translational inhibition of the target genes [51, 52]. Thus, we utilized TargetScan 7.0 [53] to explore potential KRT17 regulatory miRNAs. As a result, several conserved and less conserved miRNAs of KRT17 including miR-485-5p, miR-505-3p, miR-491-5p and miR-376-3p were predicted and were examined individually for their effects on KRT17 at mRNA level. Our results identified that the miR-485-5p was the miRNA that effectively inhibited KRT17 mRNA expression (Additional file 1: Fig. S6) and protein expression in both C9IV3 and HSC3 cells (Fig. 5A). To further verify KRT17 as a direct target of miR-485-5p and to investigate whether miR-485-5p could regulate OSCC stemness, we constructed wild-type luciferase-KRT17-3′UTR (KRT17-3′UTR-WT) plasmid and its UTR mutant plasmid (KRT17-3′UTR-MT) containing mutations on the putative miR-485-5p binding site (Fig. 5B). Data obtained from luciferase reporter assays revealed that miR-485-5p significantly reduced luciferase activity of the KRT17-3′UTR-WT reporter but not that of the mutant reporter plasmid when compared with miRNA and WT controls (Fig. 5B), suggesting that KRT17 was a direct target of miR-485-5p. Functional impacts of miR-485-5p on KRT17 were then inspected by sphere formation assay. The results demonstrated that miR-485-5p transfection significantly decreased the number of spheres formed, whereas KRT17 co-transfection rescued the inhibition of sphere formation by miR-485-5p in C9IV3 and HSC3 cells (Fig. 5C). Since chemoresistance has been the major hurdle in treating OSCC due to presence of CSCs [9, 10], we next investigated if miR-485-5p-mediated suppression of KRT17 expression and sphere formation could lead to drug sensitization. Our data revealed that drug sensitivities of the C9IV3 and HSC3 cells towards cisplatin and carboplatin were significantly enhanced in miR-485-5p-transfected cells when compared to miR-Con-transfected cells (Fig. 5D). Of note, KRT17 overexpression mildly but significantly reversed the sensitizing effects attributed to miR-485-5p (Fig. 5D). In line with these observations, miR-485-5p transfected cells elicited reduced cellular proliferation, migration and invasion, while overexpression of KRT17 significantly reversed the miR-485-5p-mediated inhibitory effects in both C9IV3 and HSC3 cells (Additional file 1: Fig. S7A–D). Furthermore, we carried out qPCR to detect miR-485-5p and KRT17 expression in 24 OSCC samples. Our results showed that the expression level of miR485-5p was negatively correlated to KRT17 expression (*p* = 0.0106) in those clinical specimens (Fig. 5E). These data corroborate with our findings from cell-based experiments and support the notion that miR485-5p negatively regulates KRT17 in OSCC. Together, our results of miR-485-5p suggest a novel role of KRT17 in maintaining OSCC stemness and drug resistance.

### Inhibition of KRT17 enhances OSCC sensitivity toward chemotherapeutics

**Fig. 6:**
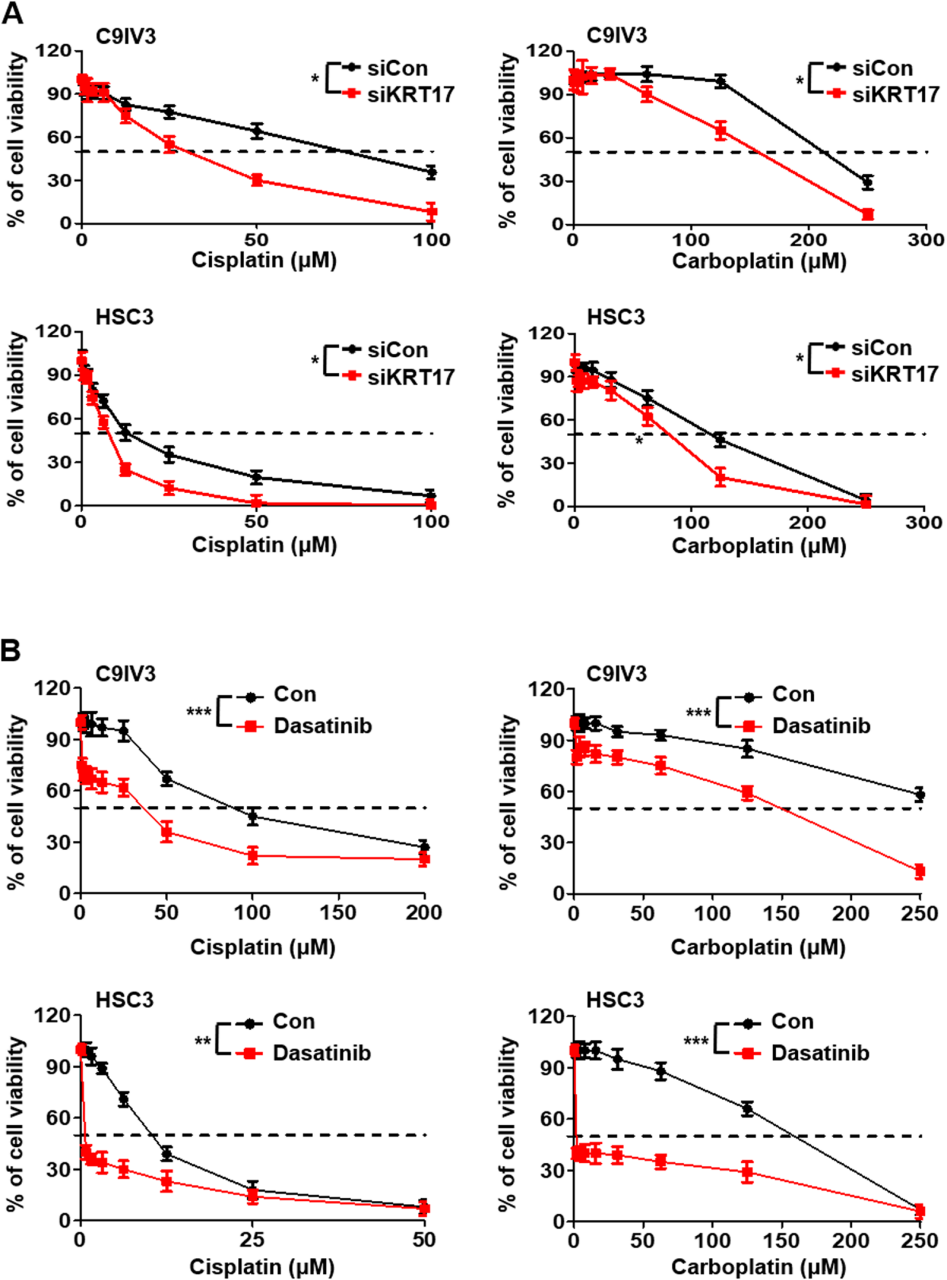
KRT17 silencing potentiates therapeutic efficacies of cisplatin, carboplatin and dasatinib in OSCC. **A** Dose-dependent cell viability assays were performed to examine the effect of KRT17 silencing on drug sensitivity of C9IV3 and HSC3 cells that were treated with cisplatin or carboplatin ranging from 0 to 100 μM. **B** Dose-dependent cell viability assays were performed to investigate whether combinatorial treatments of dasatinib with cisplatin or carboplatin could be therapeutically more effective against C9IV3 and HSC3 cells. Data are presented as the mean ± SD (*p < 0.05, **p < 0.01 and ***p < 0.001)

Since our findings in Fig. 5 indicated that depletion of KRT17 by miR485-5p elevated OSCC cellular sensitivity toward the frequently used first-line chemotherapeutics, cisplatin and carboplatin [54, 55]. To further confirm those observations, we inhibited directly KRT17 by its siRNA in C9IV3 and HSC3 cells and tested their sensitivity toward cisplatin or carboplatin. The results showed significant reduction of viabilities in RTK17 reduced cells upon the drug treatment when compared to the siCon-transfected cells (Fig. 6A). Our data showed the Src inhibitor dasatinib potently inhibited CD44 and EGFR expression through the KRT17-mediated integrin/FAK/ERK/Src signaling pathway (Fig. 4D). This led us to explore whether dasatinib could be exploited as a new chemotherapeutic for OSCC. Our results showed that dasatinib treatments significantly enhanced sensitivity towards cisplatin and carboplatin in both C9IV3 and HSC3 cells (Fig. 6B). In addition, dasatinib also potently inhibited sphere-forming abilities of the C9IV3 and HSC3 cells (Additional file 1: Fig. S8). Our data suggest that dasatinib, a clinically used drug, could potentially be exploited for treatment of KRT17 overexpressing OSCC.

### Inhibition of KRT17 sensitizes OSCC cells via suppressing cancer stemness and integrin/β-catenin signaling to impede tumor growth

**Fig. 7:**
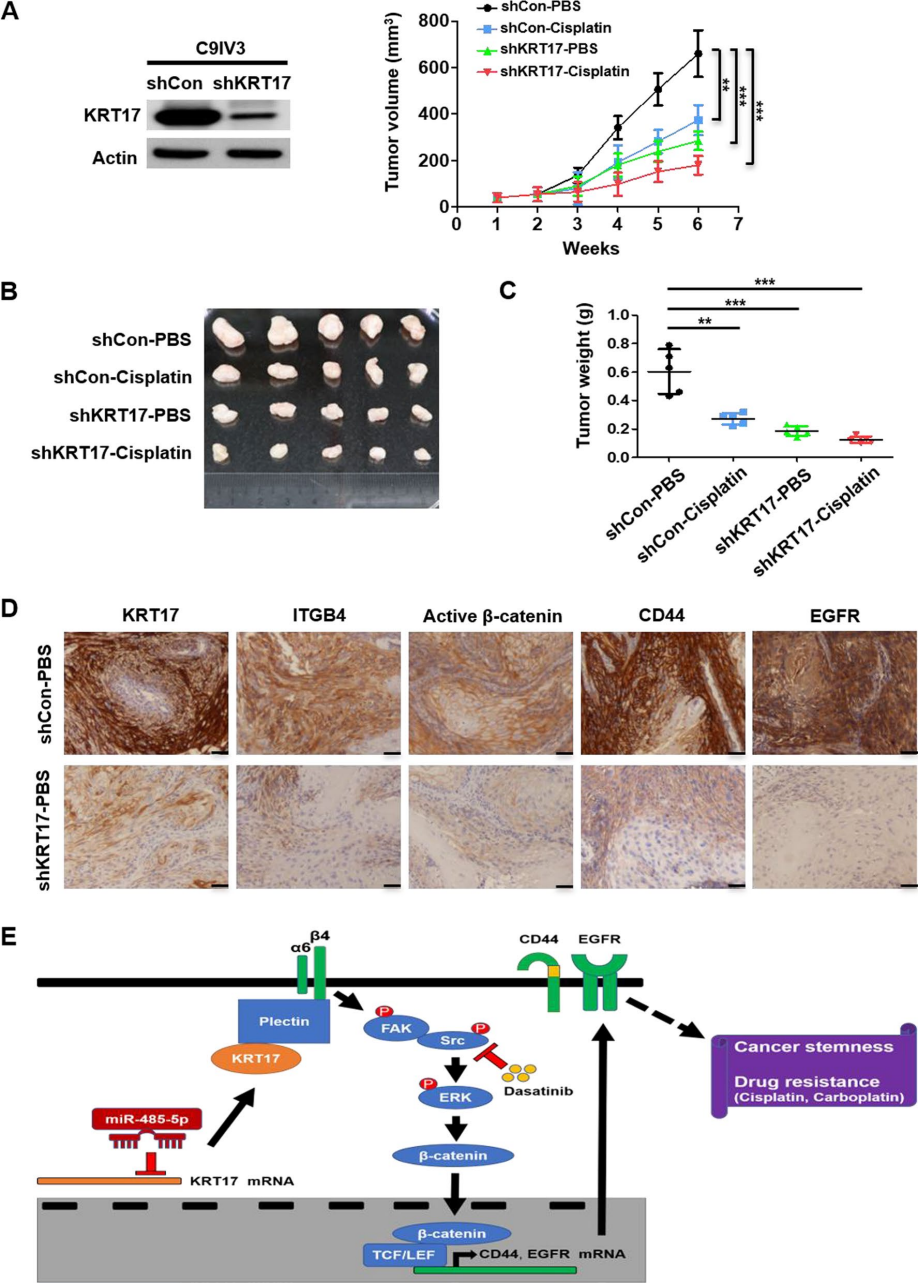
Knockdown of KRT17 inhibits tumor stemness and synergistically enhanced inhibitory effects of cisplatin on of OSCC tumor growth. **A** KRT17 knockdown (shKRT17) efficiency was determined via KRT17 protein expression by immunoblotting and comparison to the shRNA control (shCon) in C9IV3 cells (left panel). SCID mice were injected with 5 × 10^5^ shCon or shKRT17 C9IV3 cells in right flank, and PBS or cisplatin was administered two times a week. Tumor volumes were measured weekly after transimplantation before tumor excision at day 42. Right panel shows the tumor growth curve of each treatment group. Data are presented as mean ± SD (n = 5, **p < 0.01 and ***p < 0.001). **B** Excised tumors from the xenograft model was shown at the end of 42 days. **C** Tumor weights of the excised tumors (**p < 0.01 and ***p < 0.001). **D** Immunohistochemical staining for protein expressions of KRT17, ITGB4, active β-catenin, CD44 and EGFR in representative tumor excised (Scale bar, 50 μm). **E** A schematic diagram that depicts a novel signaling mechanism mediated by miR-485-5p/KRT17/integrin/FAK/Src/ERK/β-catenin that contributes to cancer stemness and drug resistance in OSCC

Our cell-based findings thus far had identified KRT17 as a novel therapeutic target and unraveled underlying mechanisms mediated by KRT17, we next pursued to demonstrate that KRT17 could be exploited as drug target in a preclinical setting. OSCC tumor-bearing mouse model was established by generating stable KRT17-knockdown C9IV3 cells by shRNA that specifically targeted KRT17 (Fig. 7A). Stable control and KRT17-knockdown C9IV3 cells were implanted into SCID mice to establish OSCC xenograft model that was subsequently treated with PBS or cisplatin. The results showed that knockdown of KRT17 or treatment with cisplatin significantly inhibited tumor growth, while cisplatin treatment in KRT17-knockdown xenografts elicited significant synergistic inhibition (Fig. 7B, C). To further corroborate with the in vitro findings described above, excised OSCC tumors were analyzed for protein expressions of KRT17, integrin β4, active β-catenin, CD44 and EGFR. IHC staining results demonstrated evident reductions in the protein expression of KRT17, ITGB4, active β-catenin, CD44 and EGFR when compared to the control PBS-treated xenograft tumors (Fig. 7D). These preclinical results thus provided a strong support for the in vitro evidence, depicting a novel miRNA-485-5p/KRT17/integrin/FAK/Src/ERK/β-catenin signaling axis that can be exploited for future drug design for cancer stem cell suppression, as well as drug sensitization in OSCC (Fig. 7E).

## Discussion

Prior to our current study, KRT17 was reported to correlate with CSCs in a few cancers such as cervical cancer and urothelial carcinoma. In cervical cancer for instance, KRT17 was induced by TGF-β1 to activate ERK1/2-MZF1 signaling pathway and enhance acquisition of CSC properties [56], while another study showed that KRT17 expression was greatly increased in cultured cervospheres and was correlated with tumorigenicity [57]. In urothelial carcinoma, KRT17 was shown to be co-expressed and co-localized with laminin receptor in highly tumorigenic cancer cell populations [58]. Nonetheless, none of these studies provided potential mechanisms that could be strategized for improving currently dismal therapeutic options available in OSCC. Hence, there is an unmet need to unveil novel mechanisms with therapeutic implication for improving the treatment of OSCC.

In this study, we have shown for the first time that KRT17 is a potent cancer stemness modulator that contributes to therapeutic resistance in invasive oral cancer cells. Our data demonstrated that KRT17 could bind to plectin, and overexpression of KRT17 increased the expression of integrin β4. In previous study, the loss of keratin 5/ keratin 14 caused dissociation of plectin and subsequent changes in β4-integrin structure that led to endocytosis [59], suggesting that KRT17 may stabilize the plectin-integrin β4 complex similar to KRT 5/14. In addition, our data showed that KRT17 affected the expression of integrins β4 and α6 proteins, which in turn activated their downstream signal transmission. Similar observations supporting our current findings were reported that arecoline treatment resulted in increased expression of KRT17 as well as integrins β4 and α6 [60], suggesting a high degree of functional association between KRT17 and integrins β4/α6. The signaling axis of integrin/FAK/Src/ERK/β-catenin modulated by KRT17 was shown to play an important regulatory role of OSCC stemness as silencing of KRT17 and β-catenin or ectopic expression of miR-485-5p consistently caused drastic reduction in CSC marker expression and sphere formation (Figs. 2, 4, 5). These findings are consistent with the manifestation of clinical oral dysplasia that was reportedly associated with greater invasiveness, metastasis, recurrence and worse survival due to cytoplasmic/nuclear delocalization of β-catenin [46, 47, 61]. We utilized β-catenin (S33Y) plasmid encoding the protein that cannot be phosphorylated and thus remains ready for nuclear translocation [62], and its overexpression led to a more efficient restoration of CD44 expression than WT β-catenin in siKRT17 treated OSCC cells (Additional file 1: Fig. S9). It could be due to the fact that WT β-catenin is easily phosphorylated by GSK3β and proceeds to proteasome-mediated degradation. In fact, β-catenin has recently been shown to upregulate stemness markers such as CD44 and EGFR in HNSCC [48–50], reassuring our observations and the therapeutic potential of targeting KRT17-mediated signaling pathway.

KRT17 has been known to form dimers with KRT6 [63]. It is not clear whether the integrin/FAK/Src/ERK/β-catenin signaling pathway that we have established is KRT6-dependent or not. To discern this, we re-investigated KRT17-mediated FAK signaling by simultaneous silencing of KRT6. Overexpression of KRT17 increased phosphorylation level of FAK by approximately 1.6-fold, whereas the inclusion of siKRT6 in KRT17-overexpressing cells led to only approximately 13% decrease when compared to KRT17 overexpression alone. Our data thus suggested that KRT17-mediated integrin/FAK/Src/ERK/β-catenin signaling axis is independent of KRT6 (Additional file 1: Fig. S10). Moreover, type 1/2 keratin dimerization were also reported to be involved in different cellular functions. In normal skin epidermis, for instance, KRT14/5 can increase AKT phosphorylation and cell cycle progression for keratinocyte. In contrast, KRT10/1 can decrease ERK1/2 phosphorylation and cell cycle progression of keratinocyte [63]. The activated AKT and ERK have been shown to enhance β-catenin signaling [45]. Thus, whether type 1/2 keratin dimerization increases or decreases cytosolic and/or nuclear β-catenin could depend on the context of cells.

The role of miR-485-5p as a tumor suppressor has recently been documented in several cancer types including breast, gastric, colorectal and ovarian cancers in inhibiting cell proliferation, invasion, metastasis, EMT and chemoresistance [64–67]. In our data, poor rescue of miR485-5p treated cells by KRT17 was found in Fig. 5D, implying possible involvement of the other targets of miR485-5p. Other regulators such as FOXK1 [68], MUC1 [69], PGC-1α [70], SRC [71] are reported to be involved in miR-485-5p-mediated inhibition of cancer cell proliferation, migration and invasion. In addition, PAK1 [72] and survivin [64] have also been shown to be associated with miR-485-5p to enhance cellular chemosensitivity. Consistently, these genes are also listed in our list of candidate targets of miR-485-5p from Targetscan 7.0. Some of those molecules aside from KRT17 could also have a role in the functional effect of miR485-5p. Interestingly, cellular chemosensitivity towards doxorubicin and paclitaxel was increased when survivin was targeted by miR-485-5p, while cisplatin resistance was claimed to be attributed to PI3K/Akt signaling targeted by miR-485-5p in ovarian cancer [64, 67]. In accordance with our observations, miR-485-5p was also recently shown to inhibit cellular migration, invasion, EMT and cisplatin chemoresistance in OSCC [72]. Therefore, our present data reveal a novel mechanism for the regulation of OSCC stemness by the miR-485-5p/KRT17/integrin/FAK/Src/ERK/β-catenin signaling axis, pointing to the option of using this miRNA coupled with effective and specific delivery vehicles as a new potential therapeutic [73].

Platinum-based chemotherapy has been the major treatment option for HNSCC, however, development of drug resistance has been the major hurdle that eventually results in tumor recurrence and dismal metastasis [9, 10, 74]. Recently, the use of Src inhibitor dasatinib for treating OSCC has been proposed as an attractive strategy via inhibiting Src downstream signaling pathway PI3K/Akt/mTOR and MEK/ERK in HNSCC [75]. Nevertheless, no significant treatment benefits were observed in HNSCC patients from an early phase 2 clinical trial on dasatinib as a single-agent [76]. Our data showing targeting KRT17 resulting in suppression of CD44 and EGFR provide a dual-targeting strategy in combination with dasatinib. A recent clinical trial revealed that combined cetuximab (EGFR-targeting) and dasatinib treatment for recurrent/metastatic HNSCC patients showed promising clinical benefits of improved OS from patients with low serum IL-6 level [77], suggesting that dual targeting of Src-EGFR could overcome drug resistance developed from cetuximab treatments. Another trial that studied EGFR inhibitor erlotinib showed significant inhibition of tumor growth, however, combination with the Src inhibitor dasatinib did not show additive effect, which could be attributed to high basal level of p-STAT3 [78]. Moreover, our observations that STAT3 was not affected in the KRT17-depleted cells that received combined dasatinib treatments with cisplatin or carboplatin showed synergistic OSCC cellular growth suppression (Figs. 3D, 6B), suggesting dasatinib could potentially be beneficial to OSCC patients with KRT17 overexpression and without STAT3 activation.

## Conclusions

In summary, our current study unraveled an important signaling cascade of miR-485-5p/KRT17/integrin/FAK/Src/ERK/β-catenin that impacts on regulation of OSCC stemness and drug resistance. Importantly, the targeting of KRT17 using its inhibitory miRNA in combination with cisplatin/carboplatin elicited synergistic therapeutic efficacy in our in vitro and in vivo OSCC models. In addition, the inclusion of Src inhibitor dasatinib in combinatorial therapies with cisplatin or carboplatin may pave the path for future therapeutics development for OSCC patients with KRT17 overexpression.

## Supplementary Information

**Additional file 1**: File contains supplementary methods, three  tables from Table S1 to Table S3 as well as figures from Figure S1 to Figure S10.

## Data Availability

All data generated or analyzed during the current study are available from the corresponding author on reasonable request.

## Abbreviations

3′UTR: 3′-Untranslated region
C9: CGHNC9
CSCs: Cancer stem cells
DEGs: Differentially-expressed genes
EGFR: Epidermal growth factor receptor
EMT: Epithelial mesenchymal transition
ERK: Extracellular signal-regulated kinase
HCC: Hepatocellular carcinoma
HNSCC: Head and neck squamous cell carcinoma
IHC: Immunohistochemistry
ITGB4: Integrin β4
KM: Kaplan–Meier
KRT17: Keratin 17
miRNA-485-5p: MicroRNA-485-5p
OSCC: Oral squamous cell carcinoma
PLA: Proximity ligation assay

## References

[1] D'Souza S, Addepalli V (2018). Preventive measures in oral cancer: an overview. Biomed Pharmacother.

[2] Rahman QB, Iocca O, Kufta K, Shanti RM (2020). Global burden of head and neck cancer. Oral Maxillofac Surg Clin North Am.

[3] Huang SH, O'Sullivan B (2017). Overview of the 8th edition tnm classification for head and neck cancer. Curr Treat Options Oncol.

[4] Naruse T, Yanamoto S, Matsushita Y, Sakamoto Y, Morishita K, Ohba S (2016). Cetuximab for the treatment of locally advanced and recurrent/metastatic oral cancer: an investigation of distant metastasis. Mol Clin Oncol.

[5] Mohan SP, Bhaskaran MK, George AL, Thirutheri A, Somasundaran M, Pavithran A (2019). Immunotherapy in oral cancer. J Pharm Bioallied Sci.

[6] Siegel RL, Miller KD, Fuchs HE, Jemal A (2021). Cancer statistics, 2021. CA Cancer J Clin.

[7] Kumar M, Nanavati R, Modi TG, Dobariya C (2016). Oral cancer: etiology and risk factors: a review. J Cancer Res Ther.

[8] Ding D, Stokes W, Eguchi M, Hararah M, Sumner W, Amini A (2019). Association between lymph node ratio and recurrence and survival outcomes in patients with oral cavity cancer. JAMA Otolaryngol Head Neck Surg.

[9] Wang C, Liu XQ, Hou JS, Wang JN, Huang HZ (2016). Molecular mechanisms of chemoresistance in oral cancer. Chin J Dent Res.

[10] Suenaga N, Kuramitsu M, Komure K, Kanemaru A, Takano K, Ozeki K (2019). Loss of tumor suppressor cyld expression triggers cisplatin resistance in oral squamous cell carcinoma. Int J Mol Sci.

[11] O'Brien CA, Kreso A, Jamieson CHM (2010). Cancer stem cells and self-renewal. Clin Cancer Res.

[12] Sarkar FH, Li Y, Wang Z, Kong D (2009). Pancreatic cancer stem cells and emt in drug resistance and metastasis. Minerva Chir.

[13] Tung SL, Huang WC, Hsu FC, Yang ZP, Jang TH, Chang JW (2017). Mirna-34c-5p inhibits amphiregulin-induced ovarian cancer stemness and drug resistance via downregulation of the areg-egfr-erk pathway. Oncogenesis.

[14] Huang W-C, Jang T-H, Tung S-L, Yen T-C, Chan S-H, Wang L-H (2019). A novel mir-365-3p/ehf/keratin 16 axis promotes oral squamous cell carcinoma metastasis, cancer stemness and drug resistance via enhancing β5-integrin/c-met signaling pathway. J Exp Clin Cancer Res.

[15] Shin K-H, Kim RH (2018). An updated review of oral cancer stem cells and their stemness regulation. Crit Rev Oncogen.

[16] Atkinson RL, Yang WT, Rosen DG, Landis MD, Wong H, Lewis MT (2013). Cancer stem cell markers are enriched in normal tissue adjacent to triple negative breast cancer and inversely correlated with DNA repair deficiency. Breast Cancer Res.

[17] Hu J, Mirshahidi S, Simental A, Lee SC, De Andrade Filho PA, Peterson NR (2019). Cancer stem cell self-renewal as a therapeutic target in human oral cancer. Oncogene.

[18] Schweizer J, Bowden PE, Coulombe PA, Langbein L, Lane EB, Magin TM (2006). New consensus nomenclature for mammalian keratins. J Cell Biol.

[19] de Pereda JM, Lillo MP, Sonnenberg A (2009). Structural basis of the interaction between integrin alpha6beta4 and plectin at the hemidesmosomes. EMBO J.

[20] Tsai F-J, Lai M-T, Cheng J, Chao SC-C, Korla PK, Chen H-J (2019). Novel k6–k14 keratin fusion enhances cancer stemness and aggressiveness in oral squamous cell carcinoma. Oncogene.

[21] Liu J, Liu L, Cao L, Wen Q (2018). Keratin 17 promotes lung adenocarcinoma progression by enhancing cell proliferation and invasion. Med Sci Monit.

[22] Kawai T, Yasuchika K, Ishii T, Katayama H, Yoshitoshi EY, Ogiso S (2015). Keratin 19, a cancer stem cell marker in human hepatocellular carcinoma. Clin Cancer Res.

[23] Khanom R, Nguyen CTK, Kayamori K, Zhao X, Morita K, Miki Y (2016). Keratin 17 is induced in oral cancer and facilitates tumor growth. PLoS ONE.

[24] Merkin RD, Vanner EA, Romeiser JL, Shroyer ALW, Escobar-Hoyos LF, Li J (2017). Keratin 17 is overexpressed and predicts poor survival in estrogen receptor-negative/human epidermal growth factor receptor-2-negative breast cancer. Hum Pathol.

[25] Wang Z, Yang M-Q, Lei L, Fei L-R, Zheng Y-W, Huang W-J (2019). Overexpression of krt17 promotes proliferation and invasion of non-small cell lung cancer and indicates poor prognosis. Cancer Manag Res.

[26] Escobar-Hoyos LF, Yang J, Zhu J, Cavallo JA, Zhai H, Burke S (2014). Keratin 17 in premalignant and malignant squamous lesions of the cervix: Proteomic discovery and immunohistochemical validation as a diagnostic and prognostic biomarker. Mod Pathol.

[27] Hobbs RP, Batazzi AS, Han MC, Coulombe PA (2016). Loss of keratin 17 induces tissue-specific cytokine polarization and cellular differentiation in hpv16-driven cervical tumorigenesis in vivo. Oncogene.

[28] Escobar-Hoyos LF, Shah R, Roa-Peña L, Vanner EA, Najafian N, Banach A (2015). Keratin-17 promotes p27kip1 nuclear export and degradation and offers potential prognostic utility. Cancer Res.

[29] Liu Z, Yu S, Ye S, Shen Z, Gao L, Han Z (2020). Keratin 17 activates akt signalling and induces epithelial-mesenchymal transition in oesophageal squamous cell carcinoma. J Proteomics.

[30] Huang WC, Chan SH, Jang TH, Chang JW, Ko YC, Yen TC (2014). Mirna-491-5p and git1 serve as modulators and biomarkers for oral squamous cell carcinoma invasion and metastasis. Cancer Res.

[31] Rhodes DR, Yu J, Shanker K, Deshpande N, Varambally R, Ghosh D (2004). Oncomine: a cancer microarray database and integrated data-mining platform. Neoplasia.

[32] Coletta RD, Yeudall WA, Salo T (2020). Grand challenges in oral cancers. Front Oral Health.

[33] Kim H, Choi GH, Na DC, Ahn EY, Kim GI, Lee JE (2011). Human hepatocellular carcinomas with "stemness"-related marker expression: Keratin 19 expression and a poor prognosis. Hepatology.

[34] Johnson DE, Burtness B, Leemans CR, Lui VWY, Bauman JE, Grandis JR (2020). Head and neck squamous cell carcinoma. Nat Rev Dis Primers.

[35] Baillie R, Tan ST, Itinteang T (2017). Cancer stem cells in oral cavity squamous cell carcinoma: a review. Front Oncol.

[36] Ribatti D, Tamma R, Annese T (2020). Epithelial-mesenchymal transition in cancer: a historical overview. Transl Oncol.

[37] Singh A, Settleman J (2010). Emt, cancer stem cells and drug resistance: an emerging axis of evil in the war on cancer. Oncogene.

[38] Wang SS, Jiang J, Liang XH, Tang YL (2015). Links between cancer stem cells and epithelial-mesenchymal transition. Onco Targets Ther.

[39] Perez SM, Brinton LT, Kelly KA (2021). Plectin in cancer: from biomarker to therapeutic target. Cells.

[40] Soung YH, Gil HJ, Clifford JL, Chung J (2011). Role of α6β4 integrin in cell motility, invasion and metastasis of mammary tumors. Curr Protein Pept Sci.

[41] Stewart RL, O'Connor KL (2015). Clinical significance of the integrin α6β4 in human malignancies. Lab Invest.

[42] Zhang S, Yu D (2012). Targeting src family kinases in anti-cancer therapies: turning promise into triumph. Trends Pharmacol Sci.

[43] Kim M, Baek M, Kim DJ (2017). Protein tyrosine signaling and its potential therapeutic implications in carcinogenesis. Curr Pharm Des.

[44] Jeong W-J, Ro EJ, Choi K-Y (2018). Interaction between wnt/β-catenin and ras-erk pathways and an anti-cancer strategy via degradations of β-catenin and ras by targeting the wnt/β-catenin pathway. NPJ Precis Oncol..

[45] Suryawanshi A, Tadagavadi RK, Swafford D, Manicassamy S (2016). Modulation of inflammatory responses by wnt/β-catenin signaling in dendritic cells: a novel immunotherapy target for autoimmunity and cancer. Front Immunol.

[46] Hu Z, Müller S, Qian G, Xu J, Kim S, Chen Z (2015). Human papillomavirus 16 oncoprotein regulates the translocation of β-catenin via the activation of epidermal growth factor receptor. Cancer.

[47] Roy S, Kar M, Roy S, Saha A, Padhi S, Banerjee B (2018). Role of β-catenin in cisplatin resistance, relapse and prognosis of head and neck squamous cell carcinoma. Cell Oncol (Dordr).

[48] Lee S-K, Hwang J-H, Choi K-Y (2018). Interaction of the wnt/β-catenin and ras-erk pathways involving co-stabilization of both β-catenin and ras plays important roles in the colorectal tumorigenesis. Adv Biol Regul.

[49] Lv X-X, Zheng X-Y, Yu J-J, Ma H-R, Hua C, Gao R-T (2020). Egfr enhances the stemness and progression of oral cancer through inhibiting autophagic degradation of sox2. Cancer Med.

[50] Warrier S, Bhuvanalakshmi G, Arfuso F, Rajan G, Millward M, Dharmarajan A (2014). Cancer stem-like cells from head and neck cancers are chemosensitized by the wnt antagonist, sfrp4, by inducing apoptosis, decreasing stemness, drug resistance and epithelial to mesenchymal transition. Cancer Gene Ther.

[51] Yoshida K, Yamamoto Y, Ochiya T (2021). Mirna signaling networks in cancer stem cells. Regen Ther.

[52] Khan AQ, Ahmed EI, Elareer NR, Junejo K, Steinhoff M, Uddin S (2019). Role of mirna-regulated cancer stem cells in the pathogenesis of human malignancies. Cells.

[53] Shin C, Nam JW, Farh KK, Chiang HR, Shkumatava A, Bartel DP (2010). Expanding the microrna targeting code: functional sites with centered pairing. Mol Cell.

[54] Cheng Y, Li S, Gao L, Zhi K, Ren W (2021). The molecular basis and therapeutic aspects of cisplatin resistance in oral squamous cell carcinoma. Front Oncol.

[55] Hanemaaijer SH, Kok IC, Fehrmann RSN, van der Vegt B, Gietema JA, Plaat BEC (2020). Comparison of carboplatin with 5-fluorouracil vs. Cisplatin as concomitant chemoradiotherapy for locally advanced head and neck squamous cell carcinoma. Front Oncol.

[56] Wu L, Han L, Zhou C, Wei W, Chen X, Yi H (2017). Tgf-β1-induced ck17 enhances cancer stem cell-like properties rather than emt in promoting cervical cancer metastasis via the erk1/2-mzf1 signaling pathway. FEBS J.

[57] Ortiz-Sánchez E, Santiago-López L, Cruz-Domínguez VB, Toledo-Guzmán ME, Hernández-Cueto D, Muñiz-Hernández S (2016). Characterization of cervical cancer stem cell-like cells: phenotyping, stemness, and human papilloma virus co-receptor expression. Oncotarget.

[58] He X, Marchionni L, Hansel DE, Yu W, Sood A, Yang J (2009). Differentiation of a highly tumorigenic basal cell compartment in urothelial carcinoma. Stem Cells.

[59] Seltmann K, Cheng F, Wiche G, Eriksson JE, Magin TM (2015). Keratins stabilize hemidesmosomes through regulation of β4-integrin turnover. J Invest Dermatol.

[60] Chiang CH, Wu CC, Lee LY, Li YC, Liu HP, Hsu CW (2016). Proteomics analysis reveals involvement of krt17 in areca nut-induced oral carcinogenesis. J Proteome Res.

[61] Reyes M, Peña-Oyarzun D, Maturana A, Torres VA (2019). Nuclear localization of β-catenin and expression of target genes are associated with increased wnt secretion in oral dysplasia. Oral Oncol.

[62] Zhou X, Zhan Z, Tang C, Li J, Zheng X, Zhu S (2020). Silencing celsr2 inhibits the proliferation and migration of schwann cells through suppressing the wnt/β-catenin signaling pathway. Biochem Biophys Res Commun.

[63] Zhang X, Yin M, Zhang LJ (2019). Keratin 6, 16 and 17-critical barrier alarmin molecules in skin wounds and psoriasis. Cells.

[64] Wang M, Cai W-R, Meng R, Chi J-R, Li Y-R, Chen A-X (2018). Mir-485-5p suppresses breast cancer progression and chemosensitivity by targeting survivin. Biochem Biophys Res Commun.

[65] Duan J, Zhang H, Li S, Wang X, Yang H, Jiao S (2017). The role of mir-485-5p/nudt1 axis in gastric cancer. Cancer Cell Int.

[66] Pan Y, Sun H, Hu X, He B, Liu X, Xu T (2018). The inhibitory role of mir-485-5p in colorectal cancer proliferation and invasion via targeting of cd147. Oncol Rep.

[67] Qiao HF, Liu YL, You J, Zheng YL, Chen LP, Lu XY (2020). G-5555 synergized mir-485-5p to alleviate cisplatin resistance in ovarian cancer cells via pi3k/akt signaling pathway. J Reprod Immunol.

[68] Liu J, Zhang J, Wang Z, Xi J, Bai L, Zhang Y (2021). Knockdown of circaplp2 inhibits progression of colorectal cancer by regulating mir-485-5p/foxk1 axis. Cancer Biother Radiopharm.

[69] Wang X, Zhou X, Zeng F, Wu X, Li H (2020). Mir-485-5p inhibits the progression of breast cancer cells by negatively regulating muc1. Breast Cancer.

[70] Lou C, Xiao M, Cheng S, Lu X, Jia S, Ren Y (2016). Mir-485–3p and mir-485–5p suppress breast cancer cell metastasis by inhibiting pgc-1α expression. Cell Death Dis.

[71] Yang Y, Liu J, Qian X, Li Y, Wang Y, Xu X (2020). Mir-485-5p improves the progression of ovarian cancer by targeting src in vitro and in vivo. Neoplasma.

[72] Lin XJ, He CL, Sun T, Duan XJ, Sun Y, Xiong SJ (2017). Hsa-mir-485-5p reverses epithelial to mesenchymal transition and promotes cisplatin-induced cell death by targeting pak1 in oral tongue squamous cell carcinoma. Int J Mol Med.

[73] Reda El Sayed S, Cristante J, Guyon L, Denis J, Chabre O, Cherradi N (2021). Microrna therapeutics in cancer: current advances and challenges. Cancers (Basel)..

[74] Yang Z, Liao J, Carter-Cooper BA, Lapidus RG, Cullen KJ, Dan H (2019). Regulation of cisplatin-resistant head and neck squamous cell carcinoma by the src/ets-1 signaling pathway. BMC Cancer.

[75] Yang Z, Liao J, Cullen KJ, Dan H (2020). Inhibition of ikkβ/nf-κb signaling pathway to improve dasatinib efficacy in suppression of cisplatin-resistant head and neck squamous cell carcinoma. Cell Death Discov.

[76] Brooks HD, Glisson BS, Bekele BN, Johnson FM, Ginsberg LE, El-Naggar A (2011). Phase 2 study of dasatinib in the treatment of head and neck squamous cell carcinoma. Cancer.

[77] Stabile LP, Egloff AM, Gibson MK, Gooding WE, Ohr J, Zhou P (2017). Il6 is associated with response to dasatinib and cetuximab: Phase ii clinical trial with mechanistic correlatives in cetuximab-resistant head and neck cancer. Oral Oncol.

[78] Bauman JE, Duvvuri U, Gooding WE, Rath TJ, Gross ND, Song J (2017). Randomized, placebo-controlled window trial of egfr, src, or combined blockade in head and neck cancer. JCI Insight.

