# Supplementary material for: MicroRNA-485-5p targets keratin 17 to regulate oral cancer stemness and chemoresistance via the integrin/FAK/Src/ERK/β-catenin pathway
Source: J Biomed Sci. 2022 Jun 15;29:42. doi: 10.1186/s12929-022-00824-z (PMC9202219; doi:10.1186/s12929-022-00824-z)
Supplement: Supplementary file 1 — Additional file 1: File contains supplementary methods, three tables from Table S1 to Table S3 as well as figures from Figure S1 to Figure S10. [file 12929_2022_824_MOESM1_ESM.docx]

**MicroRNA-485-5p targets keratin 17 to regulate oral cancer stemness and chemoresistance via the integrin/FAK/Src/ERK/β-catenin pathway**

Te-Hsuan Jang^†,1,2,3^, Wei-Chieh Huang^†,3,4^, Shiao-Lin Tung^5,6^, Sheng-Chieh Lin^3^,

Po-Ming Chen^3^, Chun-Yu Cho^2^, Ya-Yu Yang^2^, Tzu-Chen Yen^7^, Guo-Hsuen Lo^8^,

Shuang-En Chuang^*,2^ and Lu-Hai Wang^*,1,3,4^

**Supplementary Methods**

**Supplementary Table S1 to Table S3**

**Supplementary Figure S1 to Figure S10**

**Supplementary Methods**

**Transwell cell migration and invasion assay**

Cell migration was measured in 8.0-μm Falcon cell culture inserts (Corning), and for cell invasion assays, BD biocoat Matrigel invasion chamber (Corning) was used. Cells were suspended in DMEM and placed in the upper well. The bottom well was DMEM with 10% FBS. After incubating for 16 hours (migration) or 24 hours (invasion), the cells on the upper side of the insert were removed with cotton swabs, and cells at underside were stained with crystal violet. The photos of three areas were taken from insertions and analyze with Image J software.

**Soft agar assay**

C9IV3 cells were transfected with siCon or siKRT17 for 48 hours. An appropriate volume of 0.5% noble agar (Sigma) was plated in a 6-well plate and let solidified as the base agar. 1x10^5^ cells were mixed in 1ml medium with 0.5 ml 1.2% low melting agarose (Sigma) and seeded in each well on top of the base agar. Cells were cultured for 2 weeks. Colonies were stained with p-iodonitrotetrazolium violet (Sigma) for 3 days. The images were taken under a microscope and analyzed with Image J software.

**Supplementary Tables**

**Supplementary Table S1. List of siRNAs and primers used for plasmid construction**

| **The sequences of gene siRNA oligeonucleotides** | | |
| --- | --- | --- |
| **Gene** | **Sequences of siRNA oligeonucleotides** | |
| KRT17 | 1. GCCAGUACUACAGGACAAUtt |  |
|  | 2. CCCACCUGACUCAGUACAAtt |  |
|  | 3. GCGUACCAUUGUGGAAGAGtt |  |
| β-catenin | 1. CCCAAGCUUUAGUAAAUAUtt |  |
|  | 2. GGGUUCAGAUGAUAUAAAUtt |  |
|  | 3. GCCACAAGAUUACAAGAAAtt |  |
| Integrin β4 | CCAGGAAGAUCCAUUUCAAtt |  |
| Plectin | GCCAGUACAUCAAGUUCAUtt |  |
| KRT6 | CGAAGGCGUUGGACAAGUCtt |  |

| **Primers for plasmid construction** | | |
| --- | --- | --- |
| **Plasmid** | **Primer sequences (5′-3′)** |  |
| PCDNA3.1-KRT17 | F: ATATGAATTCACCATGACCACCTCCATCC |  |
|  | R: ATATCTCGAGTAGCTGAGTCCTCAGCGGG |  |
| PCDNA3.1-β-catenin | F: ATCGGGATCCGACAATGGCTACTCAAGCTGA |  |
|  | R: ATCGTCTAGAGGATGATTTACAGGTCAGTATC |  |
| pGL3-KRT17-3'UTR-WT | F: AGCTTCTAGACCCGCTGAGGACTCAGCTA |  |
|  | R: AGCTTCTAGACATAGCTGAGTCAACAAGCTT |  |
| pGL3-KRT17-3'UTR-MT | F: CTCCGGCCTCTCTAACTTTAGCCCCCTGCTTC |  |
|  | R: GAAGCAGGGGGCTAAAGTTAGAGAGGCCGGAG |  |
| **Gene** | **Sequence of shRNA oligeonucleotides** | |
| KRT17 | GCGTGACCAGTATGAGAAGAT |  |

**Supplementary Table S2. List of antibodies used for Western blot, Duolink PLA, IF, and IHC**

| **Antibodies** | **Vendor** | **Catalog number** |
| --- | --- | --- |
| CD44 | Abcam | ab157107 |
| Plectin | Abcam | ab32528 |
| β-catenin | BD | 610154 |
| Active β-catenin | Cell Signaling | 8814S |
| Integrin β4 | Cell Signaling | 14803S |
| Src | Cell Signaling | 2108S |
| p-Src(Tyr416) | Cell Signaling | 6943S |
| FAK | Cell Signaling | 3285S |
| p-FAK(Tyr397) | Gene Tex | GTX129840 |
| ALDH1A1 | Gene Tex | GTX123973 |
| Integrin α6 | Gene Tex | GTX100565 |
| Actin | Santa Cruz | sc-69879 |
| EGFR | Santa Cruz | sc-57092 |
| KRT17 | Santa Cruz | sc-393091 |
| Secondary antibody (anti-mouse) | Santa Cruz | sc-516102 |
| Secondary antibody (anti-rabbit) | Santa Cruz | sc-2357 |
| AlexaFluor 488-conjugated secondary antibody | Invitrogen | AS053 |

**Supplementary Table S3. List of primers used for RT-qPCR and RT-primer**

| **Primers used for RT-qPCR** | | |
| --- | --- | --- |
| **Gene** | **Forward primer (5′-3′)** | **Reverse primer (5′-3′)** |
| Actin | CGGCATCGTCACCAACTG | TCTCAAACATGATCTGGGTCATCT |
| ALDH1A1 | TGTTAGCTGATGCCGACTTG | CTTCTTAGCCCGCTCAACAC |
| CD133 | CACCAGGTAAGAACCCGGAT | TCAGATCTGTGAACGCCTTG |
| CD44 | GAGACAGCAACCAAGAGGCA | GTGTGGTTGAAATGGTGCTG |
| EGFR  KRT6 | CAGGAGGTGGCTGGTTATGT  CCAAGGCAGACACTCTCACA | GGACAGCTTGGATCACACTT  AGACCAAGTACGAGGAGCTG |
| KRT17 | CAACACTGAGCTGGAGGTGA | AACTTGGTGCGGAAGTCATC |
| Plectin | GGCCTCATCTGGACAATCAT | CCAGCTGGAGGTGAAGTTGT |
| Slug | GAGCATTTGCAGACAGGTCA | TCCTCATGTTTGTGCAGGAG |
| Snail | GCGAGCTGCAGGACTCTAAT | CCCACTGTCCTCATCTGACA |
| Vimentin | CAGATGCGTGAAATGGAAGA | TCCAGCAGCTTCCTGTAGGT |
| hsa-miR-376a-3p | TCATCATAGAGGAAAAT | GTGCAGGGTCCGAGGTAT |
| hsa-miR-485-5p | TCAGAGGCTGGCCGTGAT | GTGCAGGGTCCGAGGTAT |
| hsa-miR-491-5p | TCAGTGGGGAACCCTTCC | GTGCAGGGTCCGAGGTAT |
| hsa-miR-505-3p | TCCGTCAACACTTGCTGG | GTGCAGGGTCCGAGGTAT |
| RNU6B | TTCCTCCGCAAGGATGACACGC | GTGCAGGGTCCGAGGTAT |

| **Gene** | **RT-primer sequences** |  |
| --- | --- | --- |
| hsa-miR-376a-3p | GTTGGCTCTGGTGCAGGGTCCGAGGTATTCGCACCAGAGCCAACA | |
|  | CGTGG |  |
| hsa-miR-485-5p | GTTGGCTCTGGTGCAGGGTCCGAGGTATTCGCACCAGAGCCAACG | |
|  | AATTC |  |
| hsa-miR-491-5p | GTTGGCTCTGGTGCAGGGTCCGAGGTATTCGCACCAGAGCCAACC | |
|  | CTCAT |  |
| hsa-miR-505-3p | GTTGGCTCTGGTGCAGGGTCCGAGGTATTCGCACCAGAGCCAACA | |
|  | GGAAA |  |
| RNU6B | GTTGGCTCTGGTGCAGGGTCCGAGGTATTCGCACCAGAGCCAACA | |
|  | AAAATAT |  |

**Supplementary Figures**


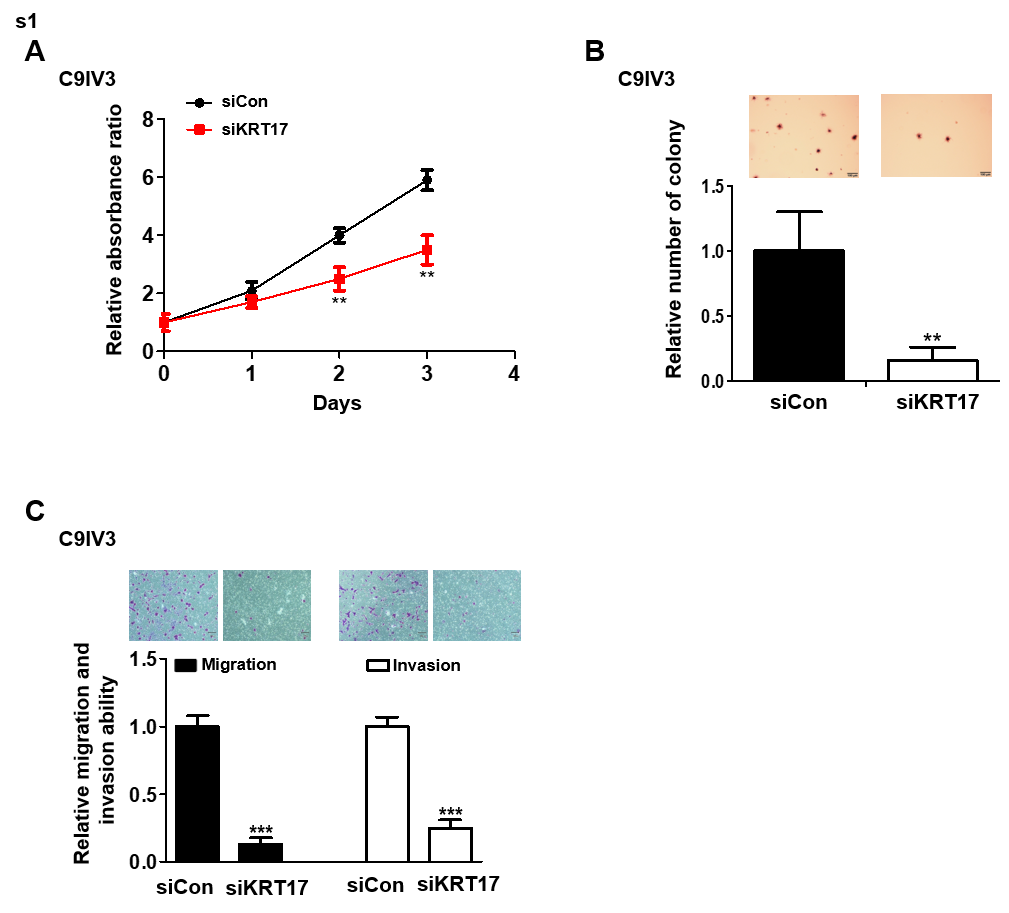


**Figure S1. Depletion of KRT17 reduces cellular proliferation, colony formation, migration and invasion abilities of C9IV3 cells.**

**A.** Cellular growth of C9IV3 cells that were transfected with siCon or siKRT17 was determined by MTS assays. **B.** Colony-forming abilities of C9IV3 cells transfected with siCon or siKRT17 were quantitatively assessed by counting number of colonies formed as described in Supplementary Methods. **C.** Migration and invasion assays were conducted as described in Supplementary Methods to evaluate KRT17-silencing effects on migration and invasion abilities of C9IV3 cells. Data are presented as the mean ± SD (*******p*<0.01 and ********p*<0.001).


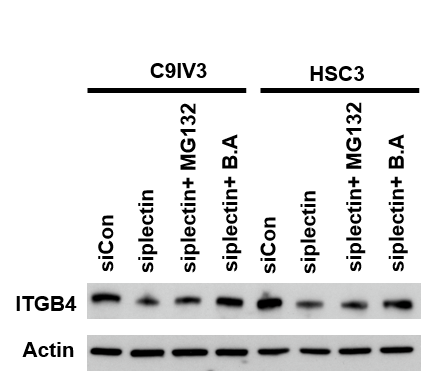


**Figure S2. Depletion of plectin increases the degradation of integrin β4 through lysosomal pathway.**

Immunoblotting was utilized to assess integrin β4 (ITGB4) protein levels in C9IV3 and HSC3 cells transfected with siCon, or siplectin. Transfected cells were then treated with 50 μM MG132 (proteasome inhibitor, Selleckchem, S2619) for 8 hours or 100 nM Baflomycin-A1 (B.A., lysosome inhibitor, Selleckchem, S1413) for 24 hours. Actin was used as the protein loading control.


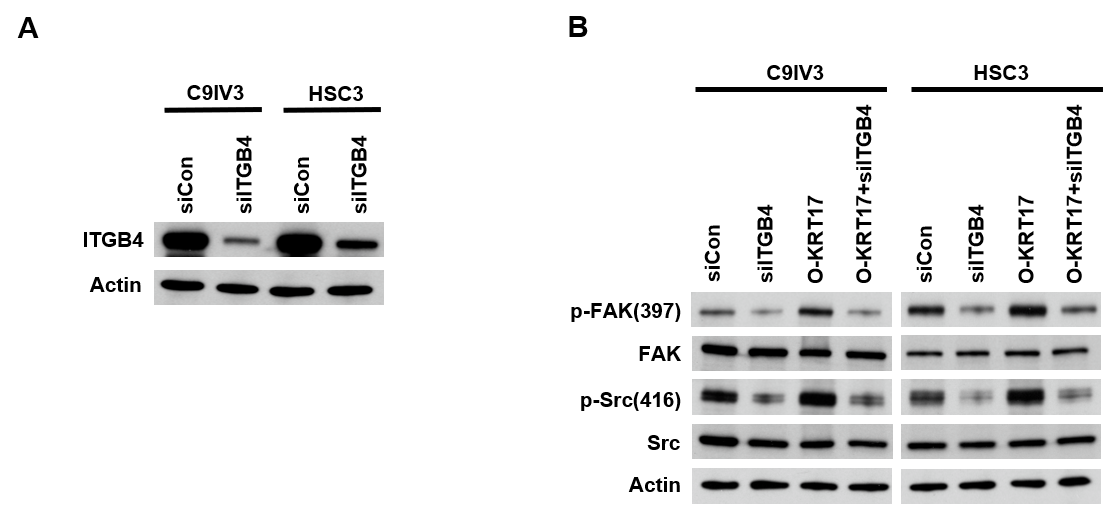


**Figure S3. Depletion of integrin β4 reduces the levels of p-FAK and p-Src in KRT17 overexpressing C9IV3 and HSC3 cells.**

**A.** siITGB4 silencing efficiencies in C9IV3 and HSC3 cells were assessed by immunoblotting that determined integrin β4 (ITGB4) protein expression. **B.** Phosphorylation levels of FAK(Tyr397) and Src(Tyr416) in C9IV3 and HSC3 cells that were transfected with siCon, siITGB4 and/or O-KRT17 (KRT17 expressing plasmid) were determined. as Actin was used as protein loading control.

0126 treatment

RD and TE671 cells were seeded at 4x105 cells/

well in 6-well plates. After 24 h, the MEK/ERK

inhibitor U0126 (Santa Cruz Biotechnology) was added

to a nal concentration of 10 μM. Following different

times of treatment, cells were collected for western blot

experiments. Mocked control cells were treated with

DMSO


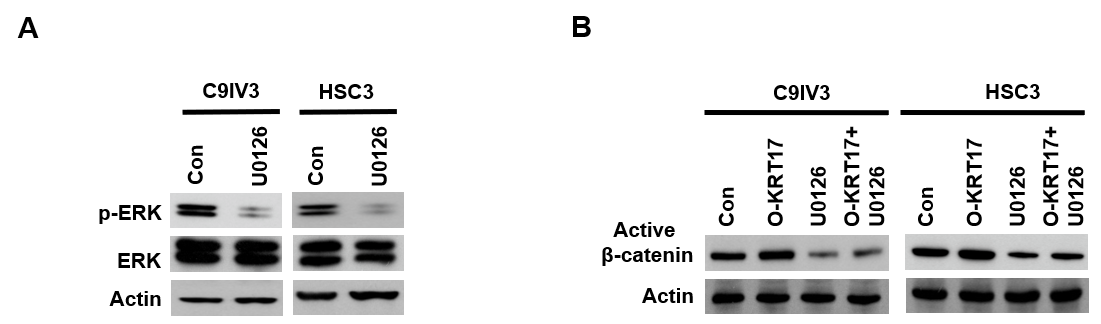


**Figure S4. Inhibition of ERK reduces the levels of active β-catenin in KRT17 overexpressing C9IV3 and HSC3 cells.**

**A.** Immunoblotting was used to assess ERK and p-ERK protein expression in C9IV3 and HSC3 cells that were treated with DMSO (Con) or 2 μM ERK inhibitor (U0126, Selleckchem, S1102) for 48 hours. **B.** Active β-catenin protein expressions in C9IV3 and HSC3 cells that were transfected with Con, or O-KRT17 plasmid for 24 hours and then treated with U0126 for 48 hours.


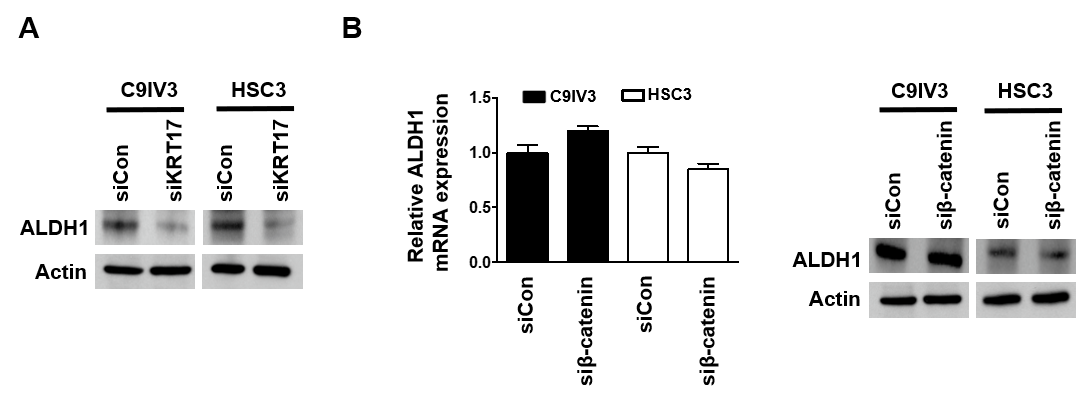


**Figure S5. Decreased ALDH1 expression after KRT17 knockdown is not due to β-catenin.**

**A.** Immunoblotting was used to assess influences of silencing KRT17 on protein expression of stemness marker ALDH1 in C9IV3 and HSC3 cells. **B.** qPCR and immunoblotting analysis were used to assess ALDH1 mRNA and protein expression, respectively, in C9IV3 or HSC3 cells that had been transfected with siCon or si-β-catenin.


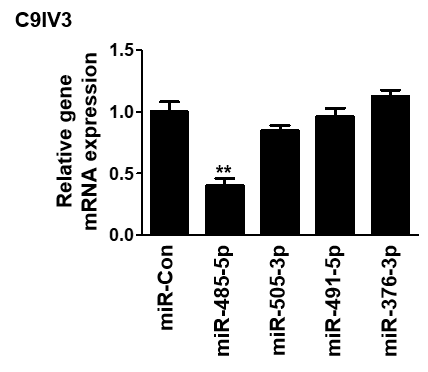


**Figure S6. miR-485-5p was identified as the miRNA that effectively suppressed KRT17 mRNA expression.**

qPCR was used to assess whether miR-Con, miR-485-5p, miR-505-3p, miR-491-5p or miR-376-3p could regulate KRT17 mRNA expression in C9IV3 cells. Data are presented as mean ± SD (******p<0.01).


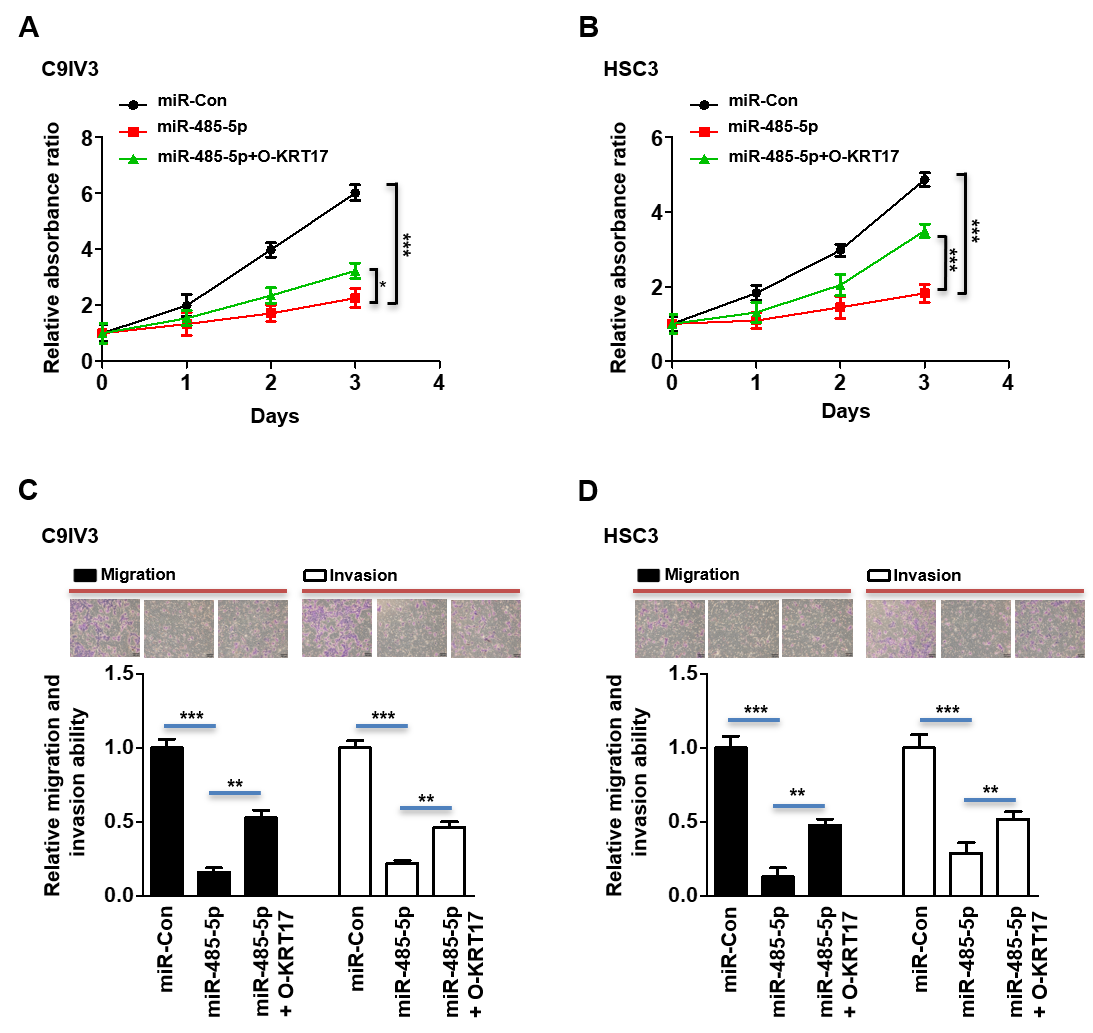


**Figure S7. Ectopic expression of KRT17 rescues cellular growth, migration and invasion that were inhibited by miR-485-5p.**

**A. B.** Cellular growth of C9IV3 and HSC3 cells transfected with miR-Con, miR-485-5p or miR-485-5p+O-KRT17 was determined by MTS assays. **C. D.** Cellular migration and invasion abilities of C9IV3 and HSC3 cells transfected with the same groups of siRNA or plasmids were determined as described in Supplementary Methods. Bar graphs show quantitative analysis of the relative abilities of migration and invasion. Data are presented as the mean ± SD (******p*<0.05, *******p*<0.01 and ********p*<0.001).


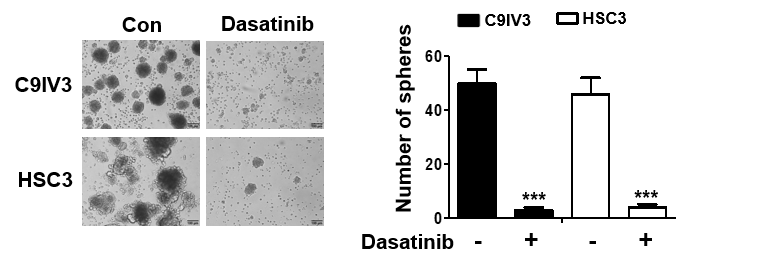


**Figure S8. Dasatinib inhibits sphere formation of the C9IV3 and HSC3 cells.**

Sphere-forming assays were conducted using C9IV3 and HSC3 cells treated with or without dasatinib. Bar graphs show the quantitative analysis of the number of spheres formed. Data are presented as the mean ± SD (********p*<0.001).


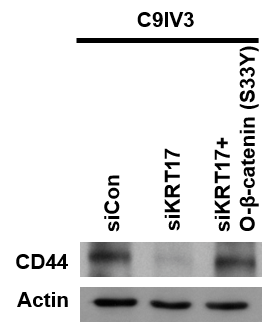


**Figure S9. KRT17 regulates CD44 expression via activation of the β-catenin signaling cascade.**

CD44 protein levels in C9IV3 cells that had been transfected with siCon, siKRT17 or siKRT17+ O-β-catenin (S33Y) plasmid were determined.


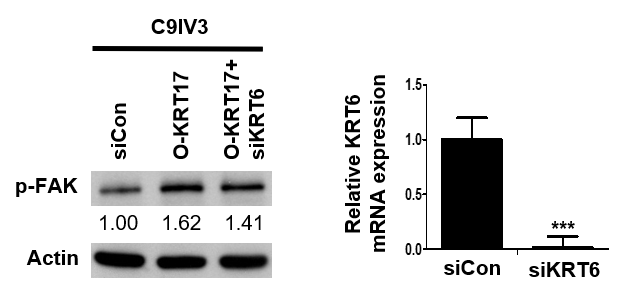


**Figure S10. Depletion of KRT6 does not significantly reduce the levels of p-FAK in KRT17 overexpressing C9IV3 cells.**

The p-FAK protein expression in C9IV3 cells that had been transfected with siCon, O-KRT17 (KRT17 overexpressing plasmid) or O-KRT17+siKRT6 was determined by immunoblotting (left). qPCR analysis was used to assess mRNA expression of KRT6 in C9IV3 cells transfected with siCon or siKRT6 (right). Data are presented as the mean ± SD (********p*<0.001).
